# Stable Gastric Pentadecapeptide BPC 157 as Therapy After Surgical Detachment of the Quadriceps Muscle from Its Attachments for Muscle-to-Bone Reattachment in Rats

**DOI:** 10.3390/pharmaceutics17010119

**Published:** 2025-01-16

**Authors:** Danijel Matek, Irena Matek, Eva Staresinic, Mladen Japjec, Ivan Bojanic, Alenka Boban Blagaic, Lidija Beketic Oreskovic, Ivana Oreskovic, Tihomil Ziger, Tomislav Novinscak, Ivan Krezic, Sanja Strbe, Martin Drinkovic, Filip Brkic, Jelena Popic, Anita Skrtic, Sven Seiwerth, Mario Staresinic, Predrag Sikiric, Ivica Brizic

**Affiliations:** 1 Department of Pharmacology, School of Medicine, University of Zagreb, 10000 Zagreb, Croatiasikiric@mef.hr (P.S.); 2 Department of Pharmacology, School of Medicine, University of Mostar, 88000 Mostar, Bosnia and Herzegovina; 3 Department of Surgery, School of Medicine, University of Zagreb, 10000 Zagreb, Croatia; 4 Department of Orthopedic Surgery, School of Medicine, University of Zagreb, 10000 Zagreb, Croatia; 5 Department of Radiology, School of Medicine, University of Zagreb, 10000 Zagreb, Croatia; 6 Department of Pathology, School of Medicine, University of Zagreb, 10000 Zagreb, Croatia

**Keywords:** pentadecapeptide BPC 157, muscle-to-bone detachment, walking recovery index (WRI), motor function index (MFI), contracture, ultrasonic imaging, MR imaging, Sirius red staining

## Abstract

Background: This is a novel rat study using native peptide therapy, focused on reversing quadriceps muscle-to-bone detachment to reattachment and stable gastric pentadecapeptide BPC 157 per-oral therapy for shared muscle healing and function restoration. Methods: Pharmacotherapy recovering various muscle, tendon, ligament, and bone lesions, and severed junctions (i.e., myotendinous junction), per-oral in particular (BPC 157/kg/day 10 µg, 10 ng), provides muscle-to-bone reattachment after quadriceps muscle detachment, both complete (rectus muscle) and partial (vastus muscles). Results: Immediately post-injury, and at 1, 2, 3, 5, 7, 14, 21, 28, 60, and 90 days post-injury, quadriceps muscle-to-bone detachment showed definitive healing failure (impaired walking and permanent knee flexure). Contrarily, macro/microscopic, ultrasonic, magnetic resonance, biomechanical, and functional assessments revealed that BPC 157 therapy recovering effects for all time points were consistent. All parameters of the walking pattern fully improved, and soon after detachment and therapy application, muscle approached the bone, leaving a minimal gap (on ultrasonic assessment), and leg contracture was annihilated. The healing process occurs immediately after detachment from both sides: the muscle and the bone. The reattachment fibers from the ends of the muscle could be traced into the new bone formed at the surface (note, at day 3 post-detachment, increased mesenchymal cells occurred with periosteum reactivation). Consequently, at 3 months, the form was stable, and the balance between the muscle and bone was the following: well-organized bone, newly formed as more cortical bone providing a narrower bone marrow space, and the muscle and mature fibers were oriented parallel to the bone axis and were in close contact with bone. Conclusions: Therefore, to achieve quadriceps muscle-to-bone reattachment, the BPC 157 therapy reversing course acts from the beginning, resolving an otherwise insurmountable deleterious course.

## 1. Introduction

This novel rat study with native peptide therapy focused on unresolved muscle-to-bone reattachment [1,2,3,4,5,6,7,8,9,10] and the stable gastric pentadecapeptide BPC 157 per-oral therapy, shared muscle healing, and function restoration [11,12,13,14].

In surgery, salvage methods include the reattachment of various muscles, i.e., quadriceps [1,2], masticatory muscles [3], lateral pterygoid muscle [4,5,6], mentalis muscle [7], and temporalis muscle [8], as well as other procedures [9,10]. Contrarily, regardless of such essential importance, up to now, the basic evidence for muscle-to-bone reattachment is scarce (for review, see [15,16,17]). So far, the experimental search of muscle reattachment following surgical detachment comprises only one monkey study, where the detached temporal muscle became (after 8 weeks) similar to its normal state in a process similar to conditions that initiate bone remodeling and bone graft replacement [18].

Thus, up to now, and despite its general importance, there has been no pharmacotherapy attempt regarding muscle-to-bone reattachment. Now, as a first attempt, we introduce BPC 157 therapy [11,12,13,14] after the dissection of the quadriceps muscle from its attachments to the femur and iliac bone in rats and, in particular, the complete dissection of the rectus muscle and partial dissection of the vastus muscles at their proximal attachments.

As already pointed out in detached temporal muscle [18] consequent to antecedent primate research [19,20,21,22], many specific points of particular tissue (muscle and bone) healing should be resolved to achieve muscle-to-bone reattachment. In general, there is a disruption of the periosteum, the vascular supply between the muscle and periosteum, and the interconnections between muscle and bone [18,19,20,21,22], and there is an elimination of the muscle tension and viscoelasticity direct effects [18]. New bone formation is absent, and an intervening connective tissue layer separates the muscle from the bone [18].

Moreover, away from the detached temporal muscle [18], the most complex circumstances can be found in the detached quadriceps muscle [23], which is an organized group of structures [23]. Certainly, this complicates and markedly reduces the possibility of resolving these disturbances to achieve muscle-to-bone reattachment. This includes bones (ilium, femur, and tibia), tendons (all four parts of the quadriceps muscle ultimately inserted into the tuberosity of the tibia via the patella, where the quadriceps tendon becomes the patellar ligament), joints, neurovascular elements, and the muscles responsible for moving the legs [23]. Thus, by using severe failure after the dissection of the quadriceps tendon from the quadriceps muscle [3] as an illustrative key, severe failure would regularly appear after the dissection of the quadriceps muscle from its attachments to the femur and iliac bone. Finally, given the wide range of motions involved in standing and walking, when re-establishing all activities of the legs, the reattachment of quadriceps muscles requires successfully coordinated actions between the quadriceps muscles and other muscle groups.

BPC 157 therapy would likely enable such coordinated action. As emphasized above, introduced as native peptide therapy, stable gastric pentadecapeptide BPC 157 therapy shared muscle healing and function restoration [11,12,13,14], largely involving striated, smooth, and heart muscle [11,12], enables the recovery of various junctions under the simultaneous healing of different particular tissues [11,12,13,14,24,25], along with other reported large beneficial effects [24,25], such as recovery of various junctions as simultaneous healing of different particular tissues [11,12,13,14,24,25]. These include restored neuromuscular junctions [26], osteotendinous junctions [27,28,29], and, in particular, restored myotendinous junctions [13]. Likewise, along with the emphasized muscle healing and functional recovery [13,26,30,31,32,33,34], it also enables the consistent healing and functional recovery of adjacent structures that would otherwise not spontaneously heal under damage. Severely damaged tendons [13,27,28,29,34,35,36,37], ligaments [38], and bones [39,40,41] can be cured. Therefore, under BPC 157 healing [11,12,13,14,24,25], bones and skeletal muscles can be integrated into functional units [42], meaning BPC 157 therapy will likely resolve complex muscle-to-bone reattachment in rats in this new follow-up study. In addition, there is a high wound-healing capability [14,24,25]. This would verify the recovery of definitive injuries, whatever noxious procedure, including crush [31,32], denervation [26,33], full transection [30,35,38], detachment [13,27,28], pseudoarthrosis [39], and bone loss [40,41]. Additionally, BPC 157 interacts with many molecular pathways [36,37,43,44,45,46,47,48,49,50,51], particularly NO molecular pathways [43,44,45], and acts as a stabilizer of cellular junctions and free radical scavengers [49,50,51].

Note that different aspects of the BPC 157 topic have been supportingly reviewed [52,53,54,55,56,57], especially musculoskeletal soft tissue healing [57].

Thus, particular advantages (i.e., being native and stable in human gastric juice even for periods longer than 24 h, allowing the absence of any carrier, and the use of various methods available for application, including via per-oral) [11,12,13,14,24,25] should be used to resolve muscle-to-bone reattachment as a native peptide therapy. BPC 157 therapy has been performed per-orally, using an effective regimen that restored the myotendinous junction [13]. Given the restored myotendinous junction [13], to approach achievable muscle-to-bone reattachment, the innate effect of BPC 157 (i.e., the successful recovery of the quadriceps muscle myotendinous junction) [13] largely departs from complex delivery (i.e., via magnetic force) [58], local application, and the use of various carriers [59,60,61,62,63]. Likewise, it overwhelms various combinations of different growth factors (i.e., transforming growth factor beta 1 (TGFβ1), insulin-like growth factor 1, and parathyroid hormone [61]; platelet-rich plasma and basic fibroblast growth factor (bFGF) [64]; and bone morphogenic protein-12 (BMP-12) and TGFβ1 [65]).

In conclusion, unlike the previously mentioned standard growth factors [61,64,65], with BPC 157 therapy introduction, there is compelling evidence that pharmacological therapy in recovering various muscle, tendon, ligament, and bone lesions and severed junctions (i.e., myotendinous junction), per-oral in particular [11,12,13,14,24,25], could also provide quadriceps muscle-to-bone reattachment following muscle dissection from its attachments to the bone. There are also supportive conceptual points providing a larger therapeutical background [24,25]. In general, there is the cytoprotection concept: the described effects of the stable gastric pentadecapeptide BPC 157 (that has been found to be safe in ulcerative colitis trials, phase II, without a lethal dose (LD1) in the toxicology studies) were ascribed to its particular cytoprotective capabilities and pleiotropic background as an acting cytoprotective peptide that protects cells and may cure muscle disturbances in particular [11,12,13,14,24,25]. As such, the cytoprotection concept initiates from the stomach [11,12,13,14,24,25]. Thus, the cytoprotection concept and the stable gastric pentadecapeptide BPC 157 (being stable, native, and originating in human gastric juice, and largely stable in water (i.e., even for 120 days)) are fully compatible with its easy applicability, including via per-oral application. This principle should be usable in muscle-to-bone reattachment in rats after dissection of the quadriceps muscle from its attachments to the femur and iliac bone. Revealing the therapy effects was in either case, macro/microscopic, ultrasonic, magnetic resonance, biomechanical, and functional, resulting in consistent muscle-to-bone reattachment.

## 2. Materials and Methods

### 2.1. Animals

This study was conducted using randomized male albino Wistar rats, 12–16 weeks of age, with a body weight of 280–320 g, under self-breeding in the Department of Pharmacology, Faculty of Medicine, Zagreb, Croatia. The facility for animals was registered by the Veterinary Directorate (Reg. No.: HR-POK-007). The laboratory rats were acclimatized for 5 days and randomly assigned to their treatment groups: 6 rats/group/interval. The laboratory animals were housed in polycarbonate (PC) cages in conventional laboratory conditions at 20–24 °C, a relative humidity of 40–70%, and a noise level of 60 dB. The cages identified the dates, study number, group, dose, number, and sex of each animal. Twelve hours of daylight were provided by using fluorescent lighting. They received standard nutrition (pelleted feed) and fresh water under free access (ad libitum) in accordance with Good Laboratory Practice (GLP). The care of the animals was in accordance with the standard operating procedures of the facility for pharmacological animals and the European Convention for the Protection of Vertebrate Animals Used for Experimental and Other Scientific Purposes (ETS 123). This research was approved by the local Ethics Committee (case number 380-59-10106-17-100/290) and by the Veterinary Directorate (UP/I-322-01/15-01/22). The ethical principles of the study are in accordance with the European Directive 010/63/E, the Act on Amendments to the Animal Protection Act (Official Gazette 37/13), the Animal Protection Act (Official Gazette 135/06), the Ordinance on the Protection of Animals Used for Scientific Purposes (Official Gazette 55/13), the recommendations of the Federation of European Laboratory Associations for Animal Science (FELASA), and the recommendations of the Ethics Committee of the Faculty of Medicine, University of Zagreb. The experiments were evaluated by an independent observer who was blind to the treatment.

### 2.2. Surgery

**Figure 1:**
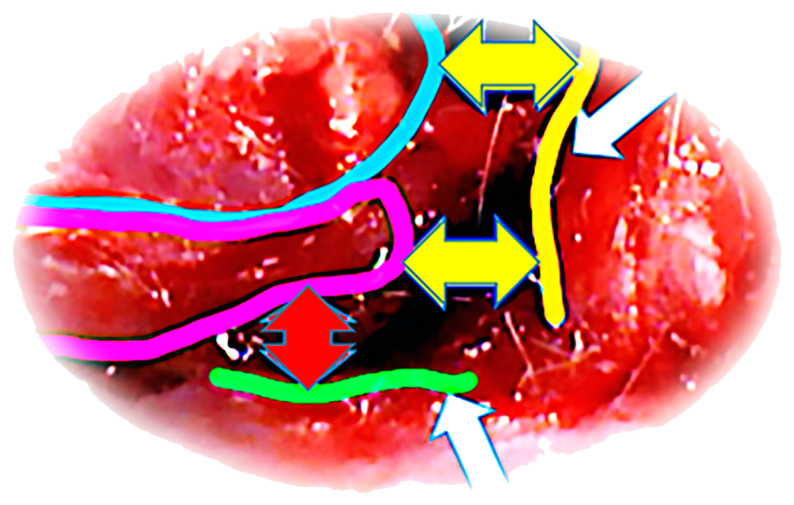
Operative approach. Display of musculature after dissection (blue line—m. rectus femoris; purple line—m. vastus lateralis). Edge of the iliac bone—yellow line (upper white arrow). Edge of the femoral bone—green line (lower white arrow). Muscle retraction distal to the iliac bone—yellow arrows (upper white arrow). Muscle retraction distal and anterior to the femoral bone—red arrow (lower white arrow).

After the administration of anesthesia (sodium thiopental; 40 mg/kg; intraperitoneally; and diazepam; 10 mg/kg; intraperitoneally) using the sterile technique, shaving the hair of the left pelvic and proximal thigh region, the rats were placed in the right lateral position (lateral decubitus position), and a longitudinal incision of 2 cm was made from the anterosuperior iliac spine to the level of the transition of the upper to the middle third of the thigh. After the skin incision, a thin layer of subcutaneous fat and muscle fascia was also incised. By performing a further careful incision, taking care not to damage the biceps femoris muscle, tensor fasciae latae, and the fascia lata itself, as well as the associated blood vessels of the region, the anatomical attachments of all four heads of the quadriceps muscles were identified and sharply dissected at their anatomical insertions with a surgical knife. The rectus femoris muscle was uncovered and completely separated from its origin on the cranial part of the anteroinferior iliac spine and supra-acetabularly [66]. In the same way, the vastus lateralis, medialis, and intermedius muscles were uncovered at the anatomical origin sites (lateral, medial, and cranial aspects of the proximal part of the femur) from which they branch off in the proximal part (Figure 1). Dissection of the three vastus muscles was performed at an average distance of 12 mm ± 3 mm measured from the apex of the greater trochanter, with an average femur length in the evaluated rats of 40 mm ± 5 mm, after which muscle retraction was observed distal to the iliac bone and distal and anterior to the femoral bone, with the appearance of a large defect between the dissected ends (Figure 1).

The skin was closed using the interrupted stitches technique with absorbable surgical sutures (Vicryl, 3.0, Ethicon Inc., Somerville, NJ, USA).

### 2.3. Drugs

As described previously [13], in muscle and tendon studies, the medication, without a carrier or peptidase inhibitor, included stable gastric pentadecapeptide BPC 157 (a partial sequence of the human gastric juice protein BPC, freely soluble in water at pH 7.0 and in saline). It was prepared as a peptide with 99% (HPLC) purity (1-des-Gly peptide was the main impurity) and manufactured by Diagen, Ljubljana, Slovenia, GEPPPGKPADDAGLV, M.W. 1419, as previously described (for review, see [11,12,13,14,24,25]).

### 2.4. Experimental Protocol

The regimen followed those described previously for functional, biomechanical, microscopical, and macroscopical assessments at the corresponding time points [13]. The medication was BPC 157 (10 µg/kg, 10 ng/kg), given per-orally, in drinking water (0.16 µg/mL; 0.16 ng/mL; 12 mL/rat/day) or drinking water (12 mL/rat/day) (controls) until they were killed, at 1, 2, 3, 5, 7, 14, 21, 28, 60, and 90 post-operative days. To assess the rats immediately following post-operative recovery and to ensure the consistency of injury based on this expected deficit in motor function, the initial application of the regimen that was used in drinking water, BPC 157; 10 µg/kg or 10 ng/kg (or water (1 mL/rat) (controls)), was given intragastrically at 5 min post-operative time.

### 2.5. Biomechanical and Functional Tests

As described previously [13], biomechanical and functional tests were performed. These included measuring the degree of shortening (contracture) of the operated leg compared to the unoperated one, calculating the walking recovery index (WRI) and the motor function index (MFI), and thigh hypotrophy by comparing the muscle diameter of the operated and non-operated side, as well as measuring the distance between the muscle fibers and anatomical insertion sites for the iliac and femoral bones. The imaging methods (ultrasound and MRI) included measuring the dynamics of the distance of the separated quadriceps muscle from the anatomical insertion points on the iliac and femoral bones.

#### 2.5.1. Walking Recovery Index (WRI)

As described previously [13], to objectively measure gait recovery, recordings were carried out using a fixed digital camera placed under the animals (Olympus mju 300 digital, Olympus Optical Co., Ltd., Hachioji, Tokyo, Japan), located 25 cm below and 12 cm to the side of a 100 cm long closed transparent plexiglass tunnel, and measurements and the gait recovery index were calculated, expressed as a coefficient of the length of the footprints with which the animal rests on the ground in certain phases of walking, according to the following Equation (1):

(1) $$WRI=\frac{NPL}{EPL}$$

where NPL stands for the non-injured animal footprint length, while EPL stands for the experimental animal footprint length, expressed in millimeters.

#### 2.5.2. Motor Function Index (MFI)

As described previously [13], to determine the motor function index (MFI), we applied a modification of the original description by Koka and Hadlock [67] (i.e., recovery of rat sciatic nerve function after transection), 1, 2, 3, 5, 7, 14, 21, 28, 60, and 90 days after surgery, in the same way as it was used in the quadriceps muscle transection study [30]. Postural thrust in extension (extension postural thrust, EPT) was measured with the rat in the upright position and indicates the mass/force with which the animal rests on the ground (using a digital scale, with an accuracy of 0.001 g) at the moment when it begins to carry its mass independently. The value of the MFI is calculated using the Equation (2):

(2) $$MFI=\frac{NEPT-EEPT}{NEPT}=1-\frac{EEPT}{NEPT}$$

where NEPT stands for thrust of the unoperated leg, EEPT stands for thrust of the experimental (operated) leg, and the value of MFI = 1 is the worst possible outcome [10]. Such an outcome occurs in situations in which the rat with the operated leg resists minimally or does not rest on the surface at all. Then, the EEPT value is close to zero, and therefore, the EEPT/NEPT coefficient tends towards zero, which brings the solution of the equation closer to (or equal to) the value of 1.

#### 2.5.3. Contracture Assessment

As described previously [13], the degree of contracture of the hind extremities was quantified to assess the functional recovery. The rats were held upright, and the degree of achieving full hip and knee extension by spontaneous and forced retraction of the hind limbs in relation to the uninjured (normal, non-operated) leg (NL) was compared. The difference was recorded as Δ (NL-EL) (in millimeters), placing a mark on the uninjured leg at the point where the big toe of the operated leg reaches.

#### 2.5.4. Ultrasonic Imaging and Measurements

The ultrasound examination was performed in rats (either deeply anesthetized for immediate post-surgery assessment or deeply sedated for assessment in later periods) with a portable ultrasound device (Philips Healthcare, Lumify, The Netherlands) by measuring the distance (in mm) of the separated muscle from the anatomical origin at the iliac and femoral bones, where the samples were placed in a predefined repeatable position, in the right lateral decubitus position with the knee and hip in full extension and both joints in the 90° flexion position.

#### 2.5.5. MRI Imaging and Measurements

The analysis of the musculoskeletal defect was also performed with the help of a 1.5 T magnetic resonance imaging (MRI) scanner (SignaTM Explorer, GE HealthCare Technologies Inc., Chicago, IL, USA). The rats were scanned 90 days after all four heads of the quadriceps femoris muscle were detached from their anatomical origins at the iliac and femoral bones. After general anesthesia, the rats were placed in a supine position with their legs naturally bent at the hips and knees. Axial scans of the pelvic and thigh regions were obtained using a T2-weighted (T2W) sequence; the images were stored and analyzed using INFINITT imaging software (Infinitt Healthcare Co., Ltd., Seoul, Republic of Korea).

### 2.6. Macroscopic Assessment

#### 2.6.1. Hypotrophy of the Thigh Musculature–Recovery of Muscle Diameter

Relative muscle atrophy was objectified by measuring the difference in quadriceps muscle diameter at the level of 1 cm distal to the apex of the large trochanter of the thigh bone, comparing the operated and non-operated side in millimeters (mm).

#### 2.6.2. Gap Assessment

After quadriceps muscle-to-bone dissection for its anatomical origins, muscle rectus femoris to the iliac bone and vastus muscle to femoral bone gaps were assessed grossly, measured by a ruler in millimeters (mm).

### 2.7. Microscopic Assessment

Microscopic assessment was carried out in a blinded fashion. The injured leg was skinned. A longitudinal cross-section of 3 mm (thickness) through the upper leg was performed, which included the whole length of the femur and adjacent muscles, enabling microscopic analysis of the whole lesion due to the right orientation of the cross-sectional slides. Before being embedded in paraffin wax, the muscle was fixed in buffered formalin (pH 7.4) for 24 h, decalcified with TBD-2 Decalcifier (ref. 6764003; Thermo Shandon Ltd., Abbey Ward, UK) for 24 h, then processed routinely to reach dehydration. Tissue samples were then embedded in paraffin wax.

Longitudinal 4 µm thick sections, including the central portion of the muscle–tendon connection, were cut and stained with hematoxylin and eosin according to the automated Sakura Tissue-Tek DRS 2000 Slide Stainer protocol (https://www.sakura.eu/Solutions/Staining-Coverslipping/H-E-Kit, accessed on 6 June 2021) following the procedural steps as follows: rehydration in distilled water; staining with hematoxylin; washing in running tap water; differentiation with 70% alcohol; staining with eosin; dehydration; clearing; and mounting. Additional sequential sections were performed using Gomori and Masson trichrome and Sirius red special histochemical staining for analysis of reticulin fibers and collagen fibers, both type 1 and type 3. Sirius red staining with polarized microscopy was conducted to evaluate the maturity of the extracellular matrix, especially secretion and production of collagen type 1, which is the major component in tendon and scarring tissue. Tissue injury was evaluated microscopically by a blinded examiner using an Olympus BX51 microscope and Olympus 71 digital camera, saving images as uncompressed 24-bit RGB TIFF files.

The tendon was scored (0–3) for collagen, vascularization, and cellularity, summarized as a total score (0–9). Collagen scoring used the following characteristics: a delicate network of small arteries oriented parallel to the collagen fibers within the thin connective septa between the bundles (score 0); less than 25% of collagen fibers of a diffuse structure with a loss of boundary between individual fibers (score 1); less than 50% of collagen fibers of a diffuse structure with a loss of boundary between individual fibers (score 2); more than 25% of collagen fibers of a diffuse structure with a loss of boundary between individual fibers (score 3). For vascularization, the following scoring was used: score 0: a delicate network of small arteries oriented parallel to collagen fibers within the thin connective septa between the bundles; score 1: less than 25% irregular vascularization and an increased number of capillaries; score 2: more than 50% irregular vascularization and an increased number of capillaries and groups of thick-walled blood vessels irregularly arranged in the hypercellular tendon; score 3: more than 50% irregular vascularization: increased number of capillaries, groups of thick-walled blood vessels irregularly arranged in the hypercellular tendon, and a nodular proliferation of blood vessels that can also be vertically directed in relation to their counterpart fibers. Cellularity used the following scoring: score 0: uniform cellularity and distribution of cells with thin wavy nuclei between collagen fibers; score 1: less than 20% higher cellularity of ribbon-oriented round nucleus cells; score 2: less than 50% higher cellularity of ribbon-oriented round nucleus cells; score 3: more than 50% higher cellularity of ribbon-oriented round nucleus cells.

### 2.8. Statistical Analysis

The statistical analyses were conducted using parametric one-way ANOVAs with post hoc Newman–Keuls tests, non-parametric Kruskal–Wallis tests, and subsequent Mann–Whitney U tests to compare groups. The values are represented as the mean ± SD as well as the minimum, median, and maximum. The results were considered significant at *p* < 0.05.

## 3. Results

With BPC 157 therapy given per-orally, from the very beginning, we revealed quadriceps muscle-to-bone reattachment in rats after surgical detachment of the quadriceps muscle from its attachments, as shown by the functional, ultrasonic, magnetic resonance, biomechanical, macroscopic, and microscopic effects assessments. In principle, by providing the special characteristics of the muscle-to-bone reattachment in rats after surgical detachment of the quadriceps muscle, these findings accord with and are supported by the healing effects so far reported on the recovery of muscles [30,31,32,33,34], tendons [27,28,29,34,35,36,37], ligaments [38], and bones [39,40,41], as well as injury and osteotendinous [27,28] and myotendinous [13] junction recovery.

### 3.1. Walking

**Figure 2:**
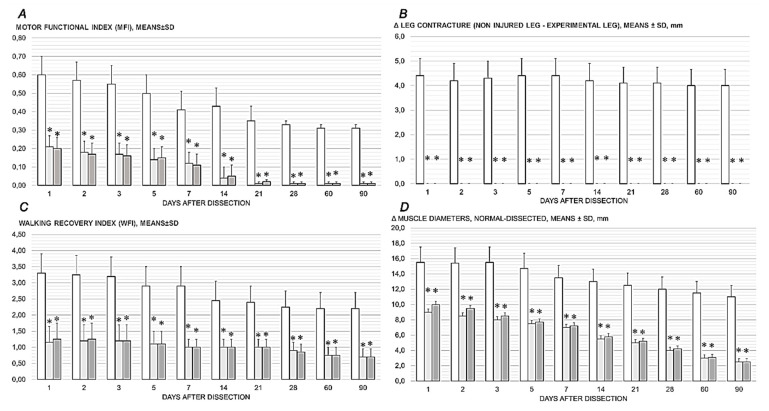
Recovering effect of BPC 157 therapy after quadriceps muscle-to-bone dissection, motor function index (MFI) (***A***), Δ leg contracture (non-injured leg–experimental leg) (***B***), walking recovery index (WRI) (***C***), Δ muscle diameters, normal-dissected (***D***). Control rats (drinking water (12 mL/rat/day), white bars), and BPC 157-treated rats (10 µg/kg daily in drinking water, light gray bars; 10 ng/kg daily in drinking water, dark gray bars) at post-surgery days 1, 2, 3, 5, 7, 14, 21, 28, 60, and 90. Mean ± SD, *—*p* ˂ 0.05, at least vs. control.

**Figure 3:**
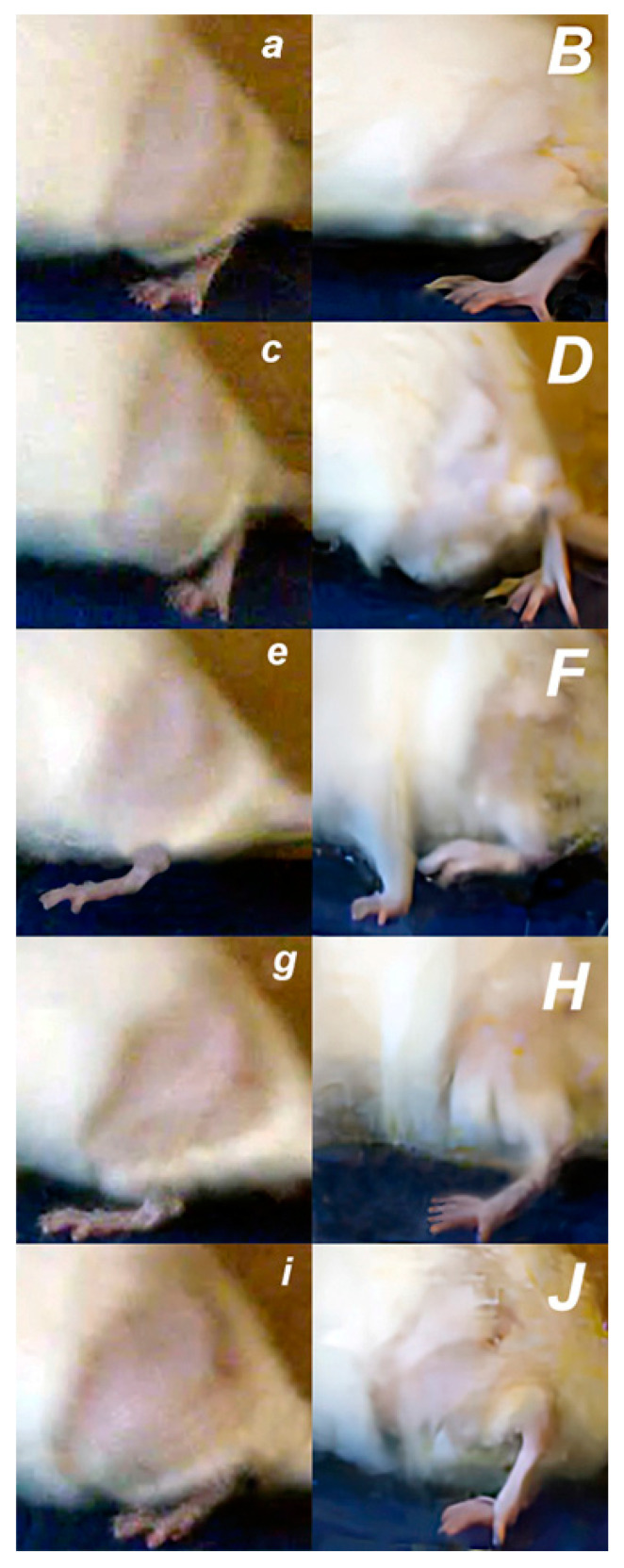
Recorded with a digital camera located to the side, recovering effect of BPC 157 therapy shown by illustrative presentation of improved walking pattern 7 days after surgery. Biomechanical gait analysis in control rats (***small italic bold letters***) and BPC 157-treated rats (***capital italic bold letters***). The gait was divided into 5 phases (initial stance phase (***a***,***B***); beginning of the swing phase (***c***,***D***); swing phase (***e***,***F***); end of the swing phase (***g***,***H***); phase of the final stance (***i***,***J***)), and particular characteristics presented as follows. (***a***,***B***) **Initiation stance phase:** Control: Foot plantar flexed, fingers flexed, knee flexed, hip flexed (***a***). Rat’s foot touching surface, partial weight-bearing. BPC 157: Foot plantar flexed, fingers extended, knee flexed, hip extended (***B***). Rat’s foot standing/supporting firmly on the surface, full weight-bearing. (***c***,***D***) **Initiation of the swing phase:** Control: Foot plantar flexed, fingers flexed, knee flexed, hip does not achieve extension (***c***). Introduction of circumduction motion of the leg with lateral foot slide and consequential pelvic tilting. BPC 157: Foot full plantar flexed, fingers extended, knee flexed, hip extended (***D***). (***e***,***F***) **Swing phase:** Control: Foot in neutral position, fingers extended, knee flexed, hip flexed (***e***). Continuation of circumduction motion of the leg with pelvic tilting. BPC 157: Foot dorsal flexed, fingers extended, knee flexed, hip flexed (***F***). (***g***,***H***) **End of swing phase:** Control: Foot in the neutral position, fingers extended, knee flexed, hip flexed (***g***). Circumduction motion of the leg with pelvic tilting. BPC 157: Foot plantar flexed, fingers extended, knee extended, hip extended (***H***). (***i***,***J***) **End stance phase:** Control: Foot in the neutral position, fingers extended, knee flexed, hip flexed (***i***). Rat’s foot touching surface, partial weight-bearing. BPC 157: Foot dorsal flexed, fingers extended, knee full flexed, hip full extended (***J***). Rat’s foot standing/supporting firmly on the surface, full weight-bearing.

**Figure 4:**
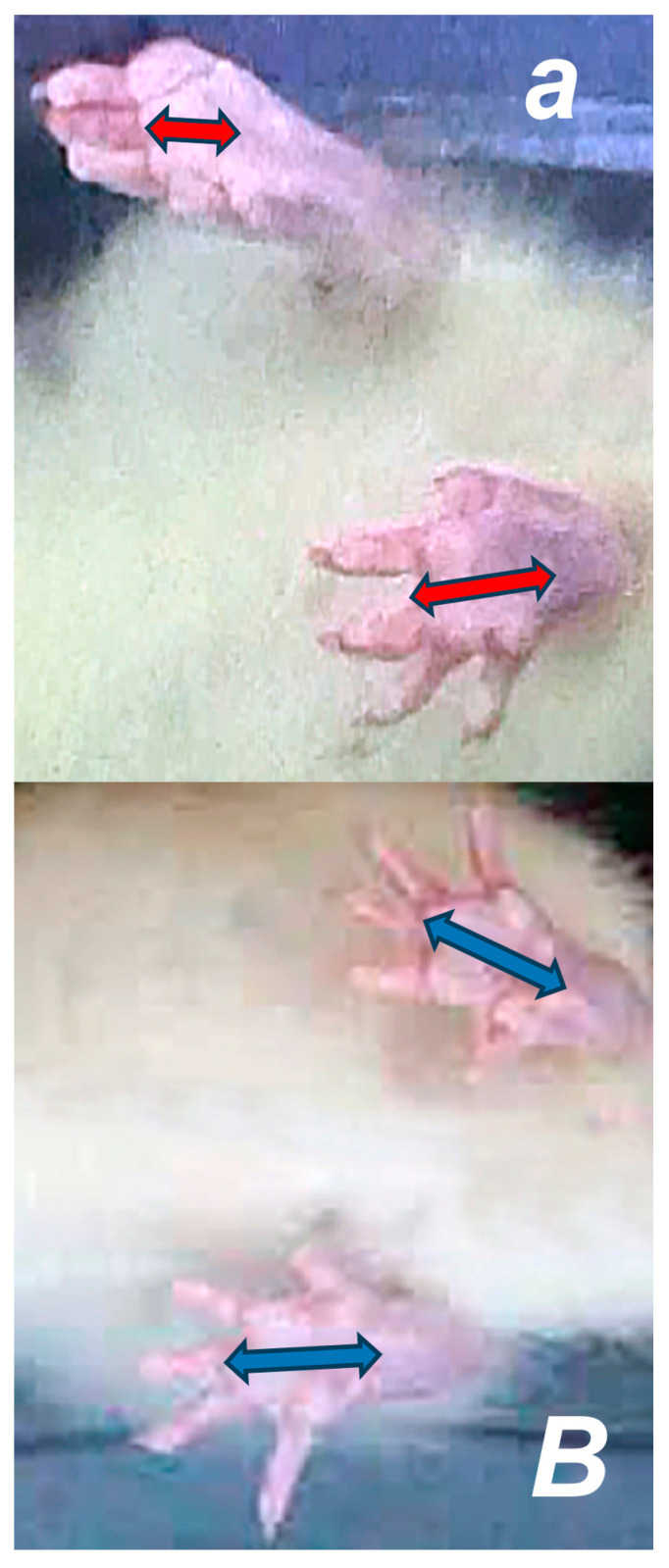
Recorded with a fixed digital camera placed under the animals’, recovering effect of BPC 157 therapy shown by illustrative presentation of improved walking pattern 7 days after surgery. Biomechanical gait analysis in control rats (***small italic bold letters***) and BPC 157-treated rats (***capital italic bold letters***). Shortening of the length of the footprint in control rats (***a***) (red arrows), and no shortening of the length of the footprint in BPC 157-treated rats (***B***) (blue arrows).

BPC 157 therapy improves walking in general (i.e., initial phase; the beginning of the swing phase; swing phase; end of the swing phase; phase of the final stance). Hip flexion failure provides that some particularities of the surgical detachment of the quadriceps muscle from its attachments consistently overwhelm disturbances following distal dissection of the quadriceps muscle from its tendon [13]. This should occur given rectus femoris muscle biarticular muscle origin (i.e., above the hip joint and below the knee joint line), with both hip flexor and knee extensor function consistently affected. Therefore, the improved walking by therapy also indicates that hip flexor and knee extensor function both consistently recovered (Figure 2, Figure 3 and Figure 4).

### 3.2. Contracture

**Figure 5:**
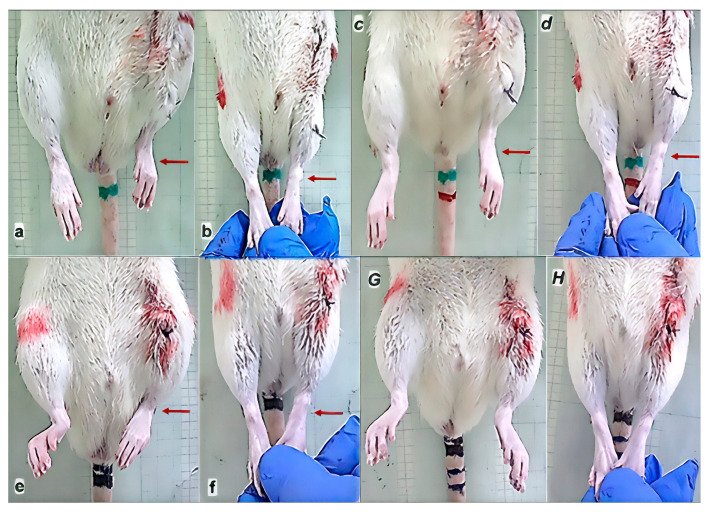
After quadriceps muscle-to-bone detachment, injured leg contracture (**a**,**b**,***c***,***d***,**e**,**f**), and the rapid recovery effect of the BPC 157 therapy (***G***,***H***) on the leg contracture. Upper, leg contracture persistent in controls (saline application), and lower, leg contracture annihilated upon BPC 157 therapy. Timeline presentation of rats before medication (left), and after medication (right). Left. Injured leg contracture (arrows) immediately after surgery (**normal small bold letters**), (**a**,**b**,**e**,**f**), contracture of the injured leg with spontaneous leg presentation (**a**,**e**), evident also upon maximal extension (**b**,**f**). Right. Presentation immediately upon medication application (intragastrically) given no change of contracture (control, right upper), or full reversal and absent contracture (BPC 157 therapy, right lower). Saline (***italic small bold letters***) (***c***, contracture (arrow),spontaneous leg presentation), (***d***, contracture (arrow) upon maximal extension). BPC 157 (***italic capital bold letters***) absent leg contracture (***G***, spontaneous leg presentation), (***H***, leg upon maximal extension); lack of arrows indicates no leg contracture (***G***,***H***)).

Dissection in the proximal part and separation of the quadriceps femoris muscle from the anatomical origin on the iliac and femoral bones reduced the pulling forces of the knee extensor and the hip flexors. This led to immediate spontaneous contracture of the injured leg, which was obvious in the upright posture. Contracture occurred whether rats were awake/anesthetized and persisted after sacrifice. Thus, contracture was permanent in the control rats and also evident with actively assisted (forced) extension. Contrarily, upon BPC 157 therapy, contracture was rapidly reversed. Therefore, an absence of leg contracture following therapy means a reduction in the pulling forces of the knee extensor and the hip flexors, and their adequate and prompt recovery in all BPC 157 rats (Figure 2 and Figure 5).

### 3.3. Motor Function Index (MFI)

Consistently, unlike the poor presentation in control rats (manifested as high MFI values), the BPC 157 rats exhibited muscle strength that had considerably recovered, given the small to negligible MFI (Figure 2).

### 3.4. Hypotrophy of the Thigh Musculature: Recovery of Muscle Diameter

**Figure 6:**
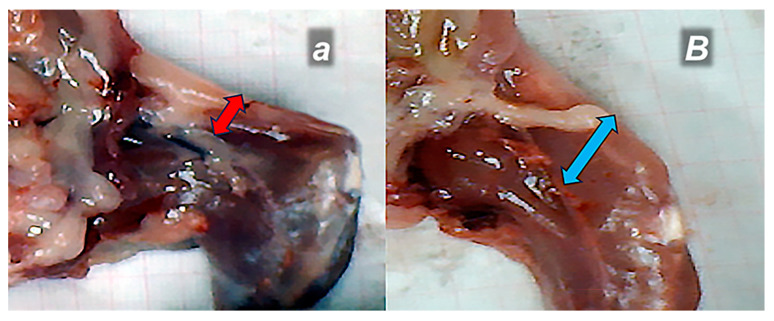
Healing improvement by BPC 157 therapy on the diameter of the quadriceps femoris muscle. Control rats (***small italic bold letters***) and in the BPC 157-treated rats (***capital italic bold letters***). At 3 months post-surgery, shortening of the diameter of the quadriceps femoris muscle in control rats (***a***) (red arrow), and no shortening of the diameter of the quadriceps femoris muscle in BPC 157-treated rats (***B***) (blue arrow).

The recovery of muscle atrophy after the traumatic event of dissection of the quadriceps femoris muscle from its bony origins was accompanied by diameter differences between the control rats and BPC 157 rats, evidencing that atrophy was persistently present in the control rats, whereas the BPC 157 rats showed clear improvement in all periods (Figure 2 and Figure 6).

### 3.5. Gap

**Figure 7:**
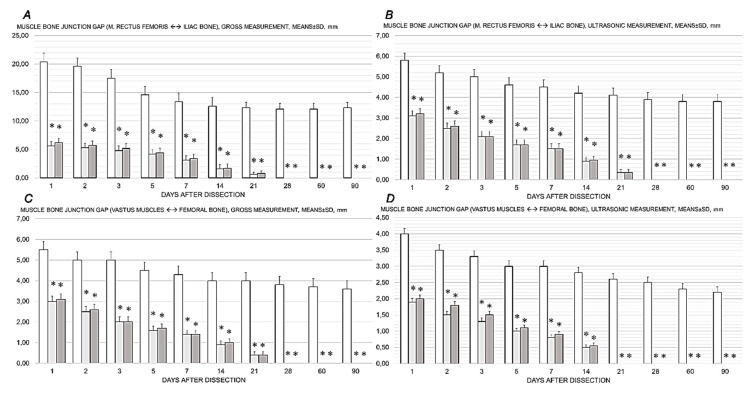
Healing improvement by BPC 157 therapy. After quadriceps muscle-to-bone dissection, the BPC 157 therapy effect goes on muscle bone junction gap, muscle rectus femoris to the iliac bone (***A***,***B***), and vastus muscle to the femoral bone (***C***,***D***), assessed grossly (***A***,***C***) or by ultrasound (***B***,***D***). Control rats (drinking water (12 mL/rat/day), white bars) and BPC 157-treated rats (10 µg/kg daily in drinking water, light gray bars; 10 ng/kg daily in drinking water, dark gray bars) at post-surgery days 1, 2, 3, 5, 7, 14, 21, 28, 60, and 90. Mean ± SD, *—*p* ˂ 0.05 at least vs. control.

**Figure 8:**
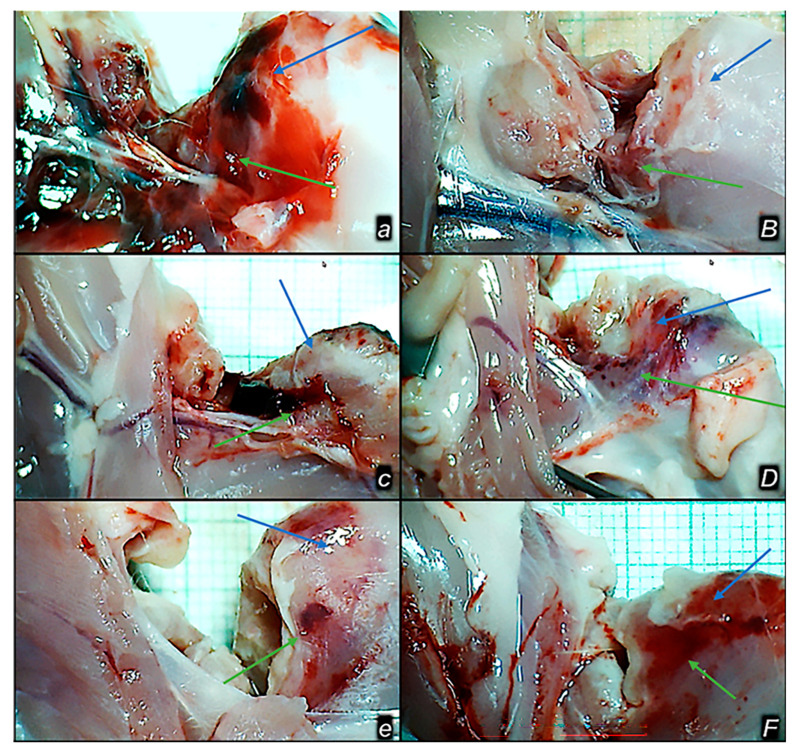
Healing improvement by BPC 157 therapy in the early post-surgery period. Persistent defect in controls (***a***,***c***,***e***) and closing upon BPC 157 therapy application (***B***,***D***,***F***). Illustrative gross presentation of the gap after quadriceps muscle dissection from bone, in rats, control (***small italic bold letters***) and BPC 157-treated (***capital italic bold letters***) on post-surgery day 1 (***a***,***B***), 2 (***c***,***D***), and 3 (***e***,***F***). M. rectus femoris (blue arrows), m. vastus (medialis) (green arrows).

**Figure 9:**
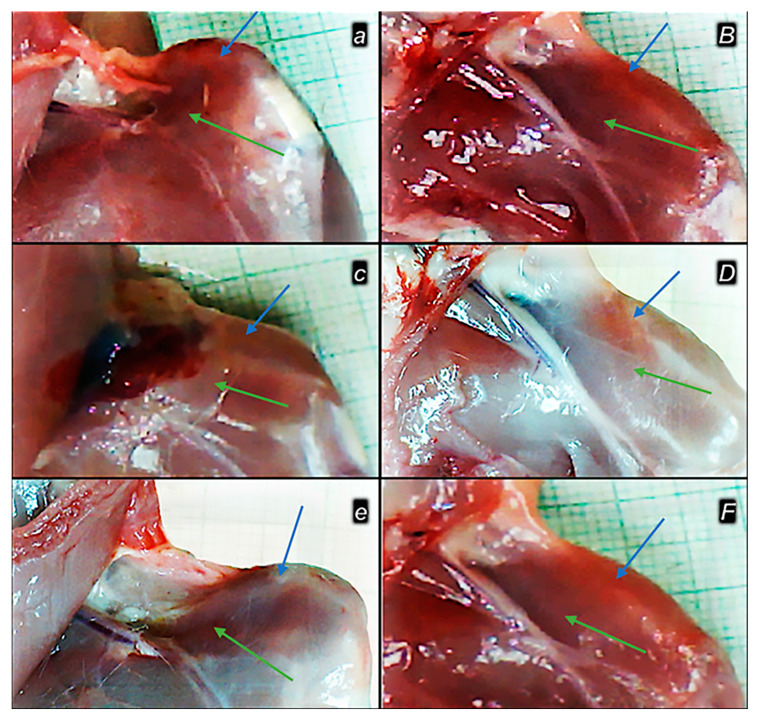
Healing improvement by BPC 157 therapy in the late post-surgery period. Persistent defect in controls (***a****,**c***,***e***) and closing upon BPC 157 therapy application (***B***,***D***,***F***). Illustrative gross presentation of the gap after quadriceps muscle dissection from bone, in rats, control (***small italic bold letters***) and BPC 157-treated (***capital italic bold letters***) on post-surgery day 28 (***a***,***B***), 60 (***c***,***D***), and 90 (***e***,***F***). M. rectus femoris (blue arrows), m. vastus (medialis) (green arrows).

**Figure 10:**
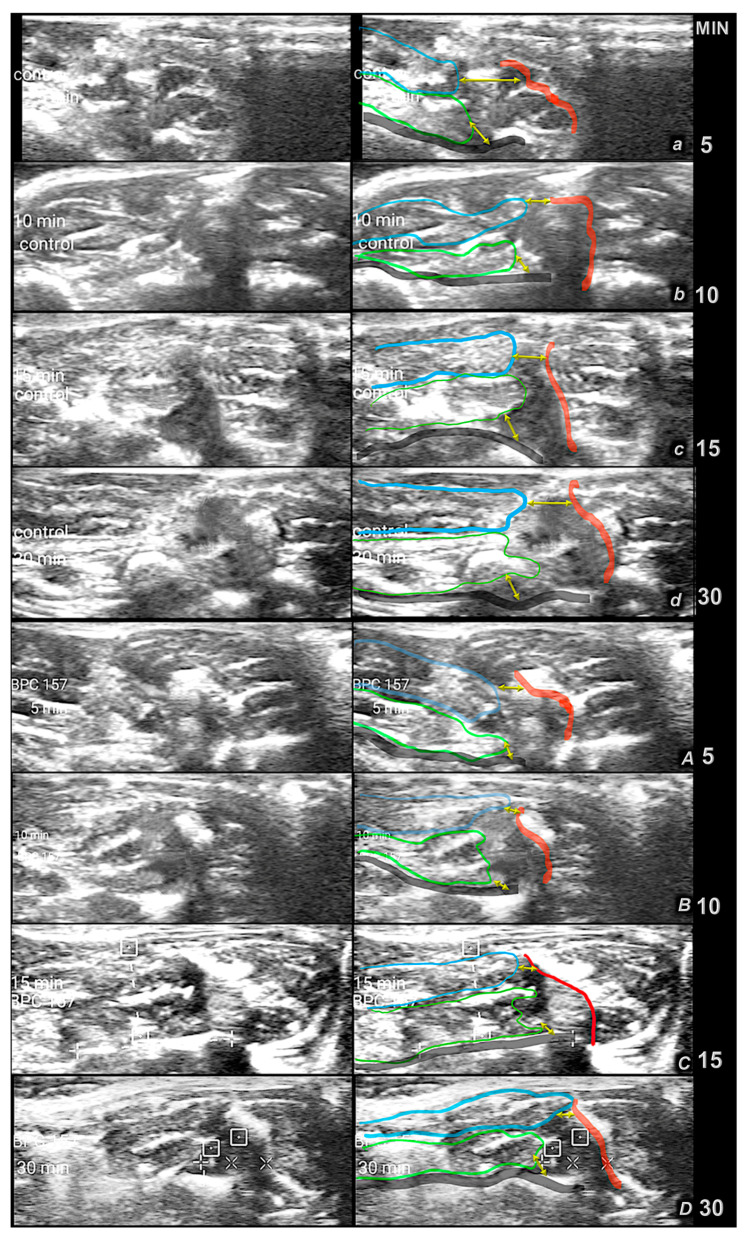
First minutes period. Ultrasound presentation of muscle bone junction gap after complete quadriceps dissection of the rat m. rectus femoris and m. vastus muscles from the anatomical origin sites at the iliac and femoral bone to illustrate the course in the first minutes, the failed course in controls (***small italic letters***), and the course toward the muscle-to-bone reattachment in BPC 157-treated rats (***capital italic letters***). Ultrasonic assessment was performed by a portable ultrasonic device (Philips Healthcare, Lumify, The Netherlands). Bone–muscle distance is expressed in millimeters, mean ± SD, after intragastric application of water in controls (5 mL/kg) (***small italic letters***) and in BPC 157-treated rats (10 µg/kg and 10 ng/kg) (***italic capital letters***) at 5 min (*a*,*A*), 10 min (*b*,*B*), 15 min (*c*,*C*), 30 min (*d*,*D*), after surgery. Yellow arrows indicate a gap. Color coding used as follows: Blue—m. rectus femoris; Green—m. vastus (medialis, intermedius, lateralis); Red—iliac bone (os ilium); Black—femoral bone (os femoris).

**Figure 11:**
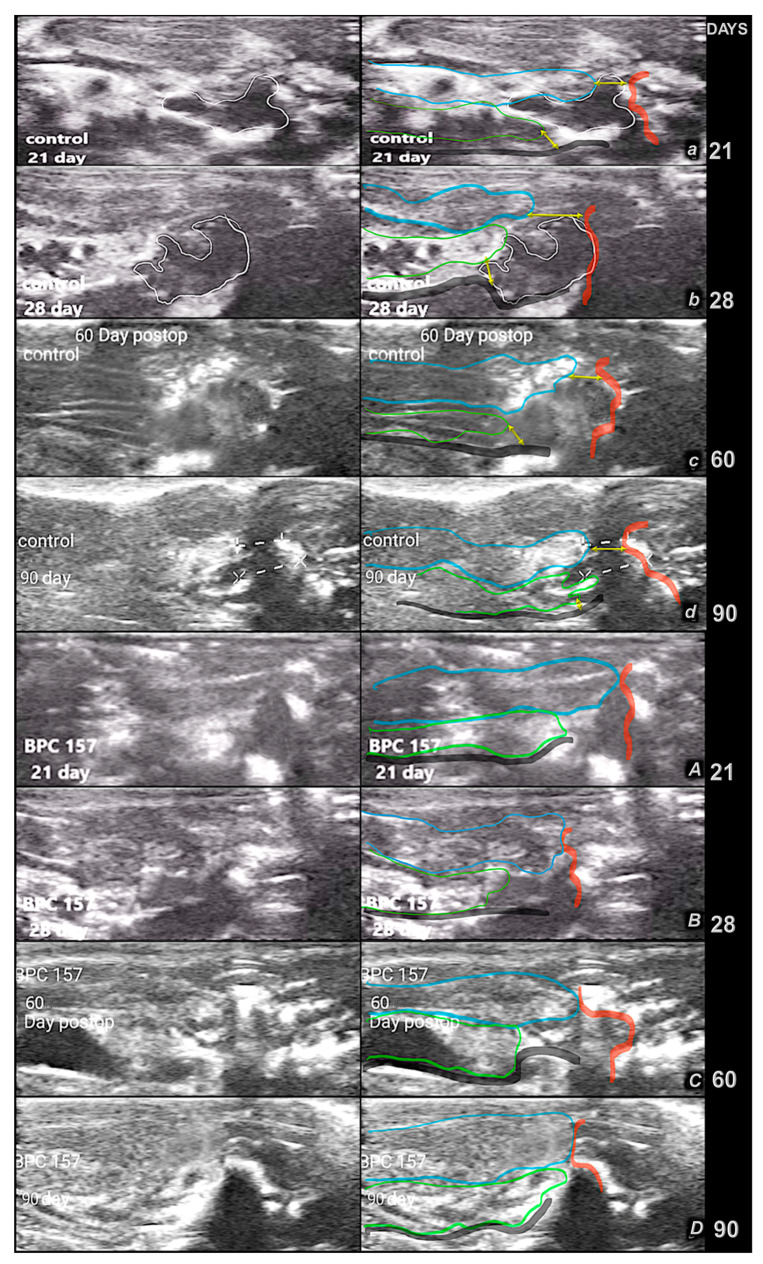
Later days period. Ultrasound presentation of muscle bone junction gap after complete quadriceps dissection of the rat m. rectus femoris and m. vastus muscles from the anatomical origin sites at the iliac and femoral bone to illustrate the course in the later days, the failed course in controls (***small italic letters***), and the course toward the muscle-to-bone reattachment in BPC 157-treated rats (***capital italic letters***). Ultrasonic assessment was performed by a portable ultrasonic device (Philips Healthcare, Lumify, The Netherlands). Bone–muscle distance is expressed in millimeters, mean ± SD, using after surgery, the application in drinking water in controls (12 mL/rat) (***small italic letters***) and in BPC 157-treated rats (10 µg/kg and 10 ng/kg) (***italic capital letters***) at 21 days (*a*,*A*), 28 days (*b*,*B*), 60 days (*c*,*C*), and 90 days (*d*,*D*). Yellow arrows indicate a gap. Color coding used as follows: Blue—m. rectus femoris; Green—m. vastus (medialis, intermedius, lateralis); Red—iliac bone (os ilium); Black—femoral bone (os femoris).

**Figure 12:**
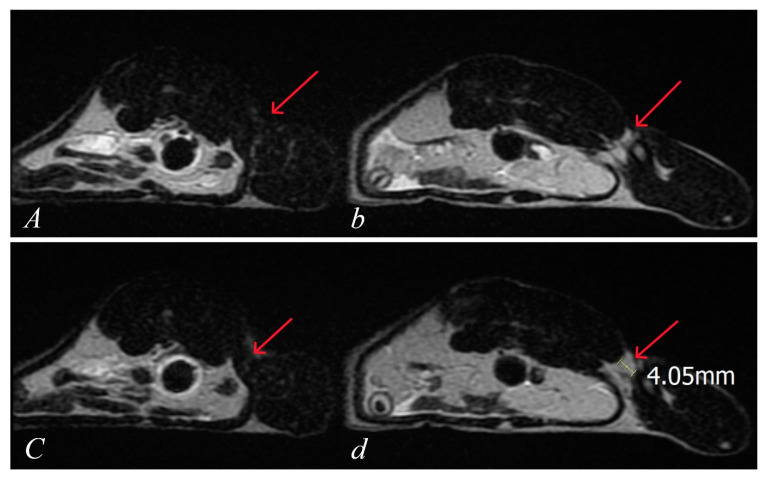
MRI imaging and measurements. Axial (***A***,***b*** *(upper level)*, ***C***,***d*** *(lower level*)) T2W images of the pelvic and upper leg region in BPC 157-treated rat (***capital italic bold letters***, ***A***,***C***) and control rats (***small italic bold letters***, ***b***,***d***) at the end of 3-month post-surgery period (1.5 T magnetic resonance imaging (MRI) scanner (Signa^TM^ Explorer, GE HealthCare Technologies Inc., Chicago, IL, USA)). The gap next to the femur due to muscle retraction in control measures 4 mm (***b***,***d***, red arrows). Attached muscle and no gap in BPC 157-treated rat (***A***,***C***, red arrows).

BPC 157 therapy was associated with a reduction in the musculoskeletal defect that had already occurred in the early stages, from the first post-operative day (Figure 7, Figure 8, Figure 9, Figure 10, Figure 11 and Figure 12). A tendency to further reduce the musculoskeletal defect was continuously present, especially after the 21st day.

In all BPC 157 rats, the defect is completely closed. Contrarily, control rats exhibited only poor healing, and persistent defect, a consistent demonstration obtained in gross macroscopy (Figure 7, Figure 8 and Figure 9).

Along with this is ultrasonic assessment (measuring the distance of the separated rectus femoris muscle from the origin point at the iliac bone and three muscles of the vastus group from the origin point at the femoral bone). In BPC 157 rats, the rectus femoris muscle reached its anatomical baseline on average after 28 days and the vastus muscle as early as 21 days after surgery (Figure 7, Figure 10 and Figure 11).

The same results were obtained for the MRI assessments. The axial scans of all control animals showed a significant distance between the threads of the retracted quadriceps muscle and the iliac bone (4.1 ± 0.5 mm), while in all rats treated with BPC 157, there was no distance, indicating the restoration of the musculoskeletal junction (*p* ˂ 0.05 at least vs. control) (Figure 12).

Likely, the distinctive achievement of complete reattachment in the rats could be due to the greater forces acting on the rectus femoris muscle, which extends over two joints (biarticular) and has two functions (auxiliary hip flexor, knee extensor), as well as the fact that this muscle is completely separated from its origin. On the other hand, the vastus muscles are only partially separated from the origin at the femoral bone, so they also had a larger active surface from which the process of reattachment began.

### 3.6. Microscopy

#### 3.6.1. HE Assessment

HE microscopy presentation reveals that quadriceps muscle detachment from its attachments is an injury that does not spontaneously recover after surgery. The failed injury course occurred in the control rats, with poor healing; these were unable to recover muscle-to-bone attachment to any extent. On the other hand, the downhill injury course was fully reversed in all BPC 157-treated rats from the very beginning, achieving muscle-to-bone reattachment to the full extent.

##### Early Period, Day 1

**Figure 13:**
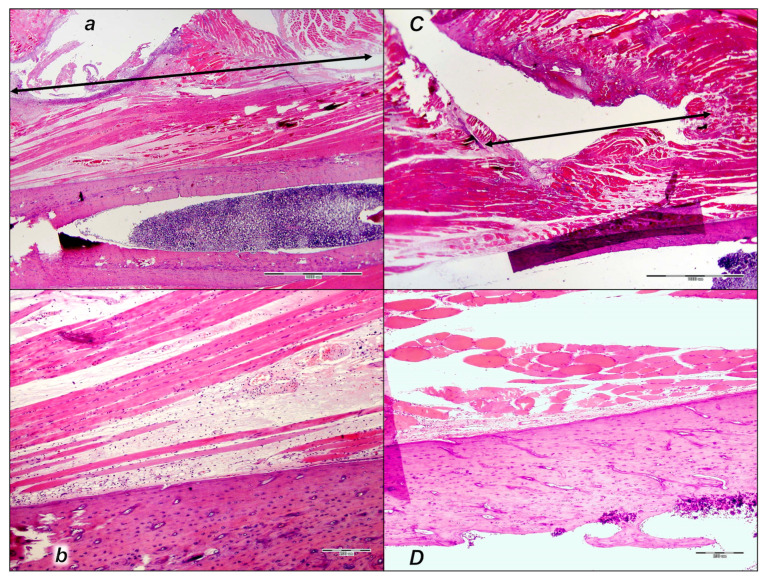
Illustrative presentation of the injury course in the control rats and failed healing (***small italic bold letters***, ***a***,***b***) vs. reversed injury course in the BPC 157-treated rats and improved healing (***capital italic bold letters***, ***C***,***D***) on day 1 after surgical detachment of the quadriceps muscle from its attachments. Control: The lesion area is larger in control (arrow), along with edema, necrosis of muscle fibers, and acute inflammation with foci of abscessation (***a***). Edema and significant separation of muscle from bone (***b***). BPC 157: The lesion is smaller (***C***) (arrow), with less edema, necrosis of muscle fibers, and acute inflammation with foci of abscessation. Minimal edema and minimal separation of muscle from bone (***D***). HE, magnification 20×, scale bar 1000 µm (***a***,***C***); HE, magnification 100×, scale bar 200 µm (***b***,***D***).

From the beginning, the control rats exhibited a large lesion area, significant edema, necrosis of muscle fibers, and acute inflammation with foci of abscessation and significant separation of the muscle from bone (Figure 13).

Contrarily, in all BPC 157-treated rats, from the very beginning, the lesion was markedly smaller (half the size, given the very large lesion in the controls), with less edema, necrosis of muscle fibers, and acute inflammation with foci of abscessation; thus, minimal edema accompanies minimal separation of the muscle from bone (Figure 13).

##### Early Period, Day 2

Consistently, as a pertinent effect, the strong distinction between the controls and the BPC 157-treated rats persisted.

**Figure 14:**
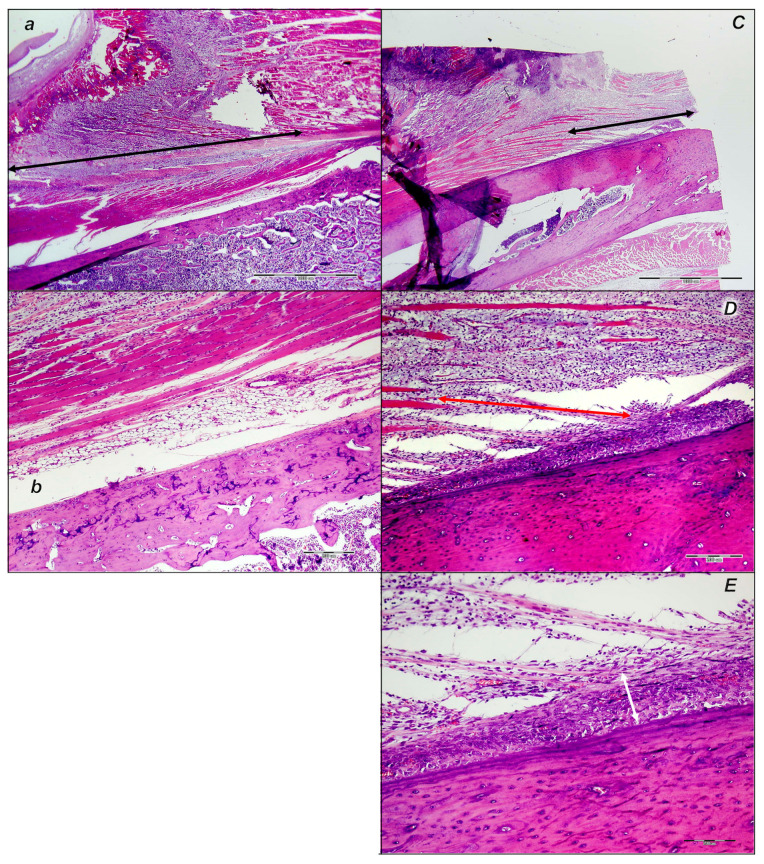
Illustrative presentation of the injury course in the control rats and further failed healing (***small italic bold letters***, ***a***,***b***) vs. reversed injury course in the BPC 157-treated rats and further improved healing (***capital italic bold letters***, ***C–E***) on day 2 after surgical detachment of the quadriceps muscle from its attachments. Control: The lesion area is larger in control, along with edema, necrosis of muscle fibers, and acute inflammation with foci of abscessation (***a***) (half of the lesion is shown, black arrow). At the border of bone tissue and muscle, significant edema, no reaction of periosteum, mesenchymal stem cells (***b***). BPC 157: The lesion is smaller (***C***), with less edema, necrosis of muscle fibers, and acute inflammation with foci of abscessation (half of the lesion is shown, black arrow). At the border of bone tissue, periosteum reaction, close contact with muscle fibers (red arrow) (***D***), and proliferation of mesenchymal stem cells (white arrow) (***E***). HE, 20×, scale bar 1000 µm (***a***,***C***); HE, magnification 40×, scale bar 200 µm (***b***,***D***); HE, magnification 200×, scale bar 200 µm (***E***).

In the control rats with a large lesion area and at the border of bone tissue and muscle, there was significant edema and no reaction of periosteum for mesenchymal stem cells (i.e., on post-operative day 2) (Figure 14).

##### Early Period, Day 3 to Day 7

**Figure 15:**
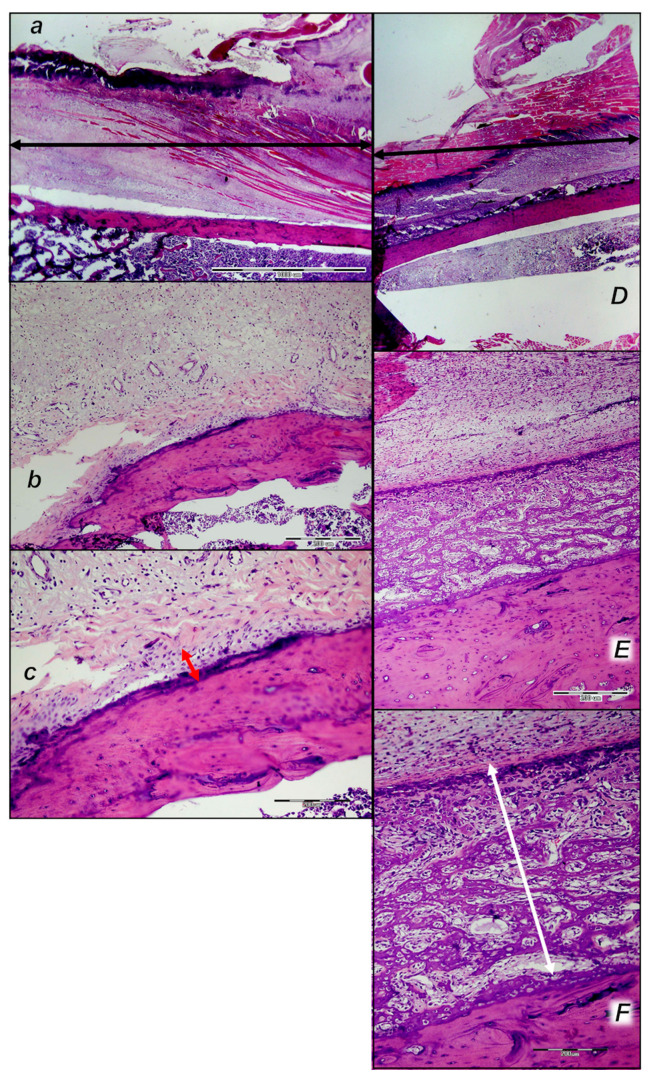
Quadriceps muscle-to-bone detachment, post-surgery day 7 (corresponding to the presentation on day 3 and day 5), injury course in the control rats and further failed healing (***small italic bold letters***, ***a***–***c***) vs. reversed injury course in the BPC 157-treated rats and further improved healing (***capital italic bold letters***, ***D***–***F***). Control: The lesion area is larger in control, along with edema, necrosis of muscle fibers, and acute inflammation with foci of abscessation (***a***) (half of the lesion is shown, black arrow). At the border of bone tissue and muscle, there was significant edema, minimal reaction of periosteum, mesenchymal stem cells, and rare and irregularly organized cells towards loose connective, granulation (***b***,***c***, red arrow). BPC 157: The lesion is smaller (***D***), with moderate edema, necrosis of muscle fibers, and acute inflammation with foci of abscessation (half of the lesion is shown, black arrow). At the border of the bone tissue, the reaction of the periosteum was visible, the formation of bone tissue was organized, the diameter of the bone was larger, and the proliferation of mesenchymal stem cells (***E***,***F***, white arrow) and close contact with the muscle fibers were still observed on the surface. HE, magnification 20×, scale bar 1000 µm (***a***,***D***); HE, magnification 100×, scale bar 200 µm (***b***,***E***); HE, magnification 200×, scale bar 200 µm (***c***,***F***).

Likewise, controls have a minimal reaction of periosteum for mesenchymal stem cells, as well as rare and irregularly organized cells towards loose connective granulation (in the post-operative period from day 3 to day 7) (Figure 15).

Contrarily, at all of these early time points, a strong reversal occurred in BPC 157-treated rats (Figure 14 and Figure 15). The lesion was smaller, with less edema, less necrosis of muscle fibers, and less acute inflammation with foci of abscessation. Noteworthy, at the border of bone tissue, there was a periosteum reaction and close contact with muscle fibers, and the proliferation of mesenchymal stem cells occurred (Figure 14 and Figure 15).

##### Later Period, Period Until Post-Operative Day 21

Likewise, in the later period (in the post-operative period until post-operative day 21), the poor healing course in the controls and advanced reversal in the BPC 157-treated rats consistently occurred.

**Figure 16:**
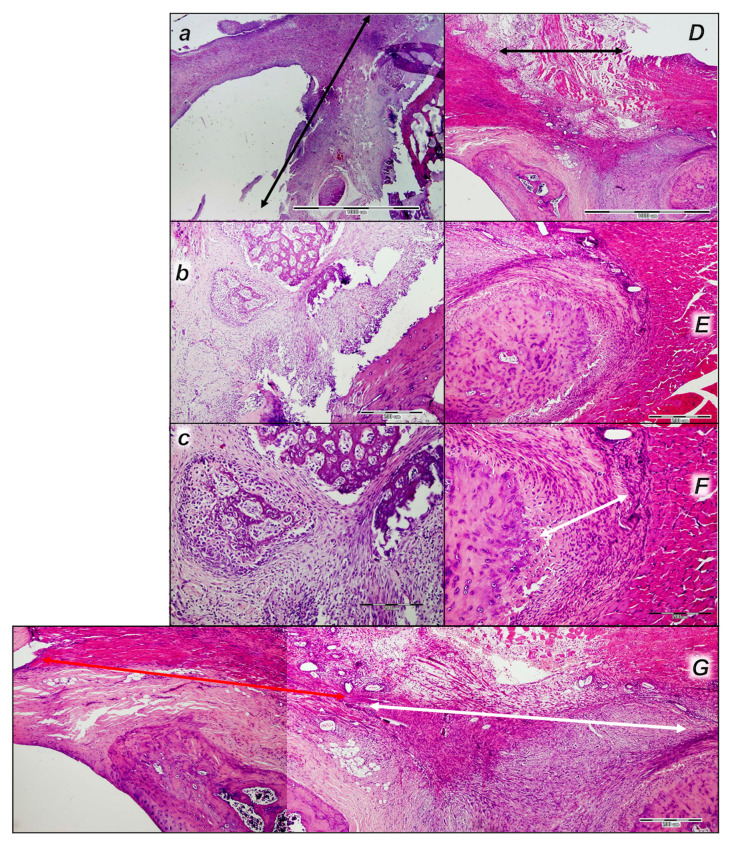
Quadriceps muscle-to-bone detachment, post-surgery day 14 (corresponding to the presentation on day 21), injury course in the control rats and further failed healing (***small italic bold letters***, ***a***–***c***) vs. reversed injury course in the BPC 157-treated rats and further improved healing (***capital italic bold letters***, ***D***–***G***). Control: The lesion area is larger in control, along with edema, necrosis of muscle fibers, and acute inflammation with foci of abscessation (***a***) (half of the lesion is shown, black arrow). At the border of bone tissue and muscle, granulation tissue, disorganized formation of bone tissue, and islands of bone tissue (presumed appositional growth), without contact with bone, were observed (***b***,***c***). BPC 157: The lesion is smaller (***D***), with moderate edema, muscle fibers necrosis, acute inflammation, and no foci of abscessation (half of the lesion is shown, black arrow). At the border of the bone tissue, a periosteal reaction was observed, organized formation of bone tissue, the diameter of the bone was larger, and the proliferation of mesenchymal stem cells (white arrow) and close contact with muscle fibers (red arrow) were still present on the surface (***E***–***G***). HE, magnification 20×, scale bar 1000 µm (***a***,***D***); HE, magnification 100×, scale bar 200 µm (***b***,***E***,***G***); HE, magnification 200×, scale bar 200 µm (***c***,***F***).

In the controls, the lesion area was still large, along with edema, necrosis of muscle fibers, and acute inflammation with foci of abscessation. Moreover, at the border of bone tissue and muscle, there was granulation tissue, disorganized formation of bone tissue, and islands of bone tissue (presumed to be appositional growth) without contact with bone (Figure 16).

Contrarily, in post-operative period until post-operative day 21, in the BPC 157 rats, the lesion was smaller, with moderate edema, necrosis of muscle fibers, and acute inflammation with foci of abscessation (Figure 16). At the border of the bone tissue, the reaction of the periosteum was visible, the formation of bone tissue was organized, the diameter of the bone was larger, and the proliferation of mesenchymal stem cells and close contact with the muscle fibers were still observed on the surface (Figure 16).

##### Later Period, Final Post-Operative Month Periods

**Figure 17:**
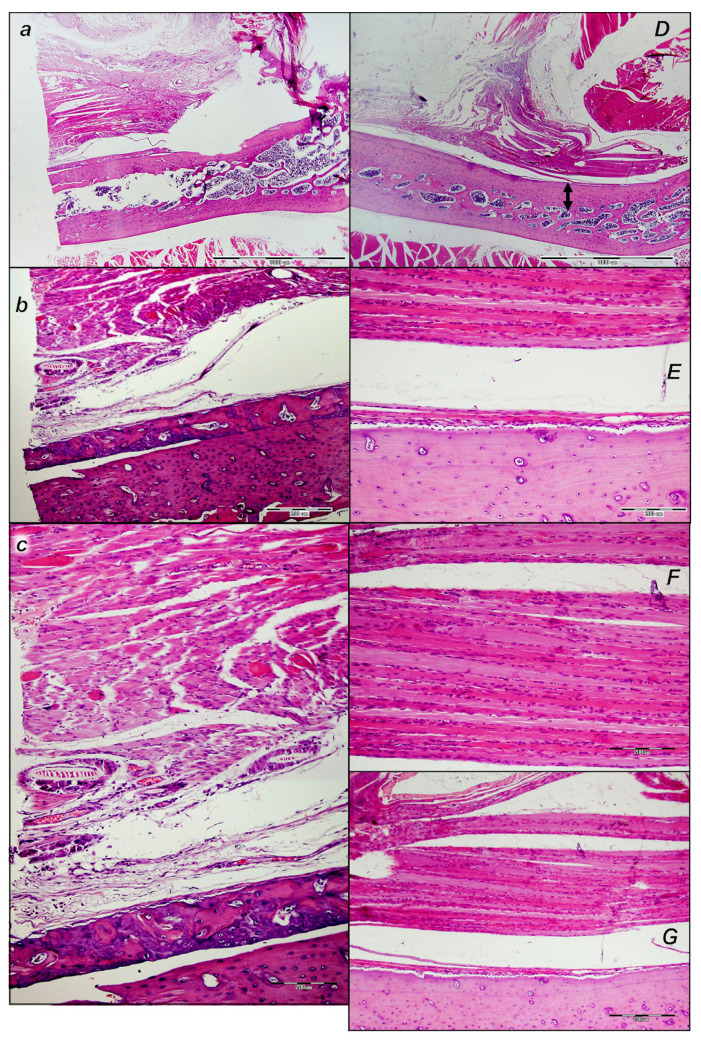
Quadriceps muscle-to-bone detachment, 1-month post-surgery (corresponding to the presentation at 2 months), injury course in the control rats and further failed healing (***small italic bold letters***, ***a***–***c***) vs. reversed injury course in the BPC 157-treated rats and further improved healing (***capital italic bold letters***, ***D–G***). Control: A loose connective tissue was observed at the border of bone tissue and muscle, there was no close contact with muscle fibers, nor was there any organized formation of bone tissue, but there were islands of bone tissue. The muscle fibers appeared like immature fibers, while mature fibers are very few (red fibers) (***a***–***c***). BPC 157: Minimal bone tissue activity was observed in the lesion area; the bone tissue was well organized and in close contact with muscle fibers that were mature in appearance and adequately oriented, parallel to the axis of the bone. The newly created bone evidenced the scarcer bone marrow in the treated animal compared to the cortex in the control animals (black arrows). The scarcer bone marrow in the treated animals compared to the thickness of the cortex in the control animals was the main feature of the newly created bone (black arrows). HE, 20×, scale bar 1000 µm (***a***,***D***); HE, magnification 100×, scale bar 200 µm (***b***,***E***); HE, magnification 200×, scale bar 200 µm (***c***,***F***,***G***).

**Figure 18:**
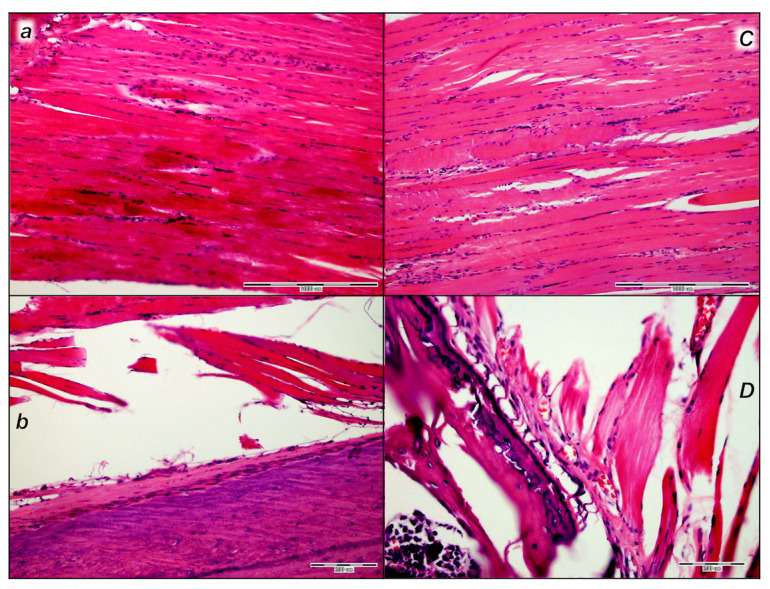
Quadriceps muscle-to-bone detachment, 3-months post-injury course in the control rats and further failed healing (***small italic bold letters***, ***a***,***b***) vs. reversed injury course in the BPC 157-treated rats and further improved healing (***capital italic bold letters***, ***C***,***D***). The appearance of the muscle fibers and the contact of the muscle fibers with the bone were identical in appearance to the 1-month-old treated animals. The muscle fibers were more mature, especially those fibers in contact with the bone (observed muscle fibers next to the bone in the control were thin). HE, magnification 20× (***a***,***C***); HE, magnification 100× (***b***,***D***).

In the final post-operative month periods (Figure 17 and Figure 18), at the border of bone tissue and muscle, the control rats exhibited loose connective tissue and no close contact with muscle fibers and absent organized formation of bone tissue, but islands of bone tissue consistently appeared (Figure 17 and Figure 18). The muscle fibers looked like immature myofibers, and very few mature myofibers (red fibers) occurred.

On the other hand, in the final post-operative month periods (Figure 17 and Figure 18), in the BPC 157 rats, the bone tissue of the lesion was minimally active and well organized. There was close contact with mature muscle fibers that were well-oriented parallel to the axis of the bone. The evidence of new bone formation covered a smaller area of the bone marrow, at the expense of the formation of cortical bone in the treated animals compared to the cortex in the control animals.

#### 3.6.2. Analysis of the Injured Area with Special Histochemical Sirius Red Staining

**Figure 19:**
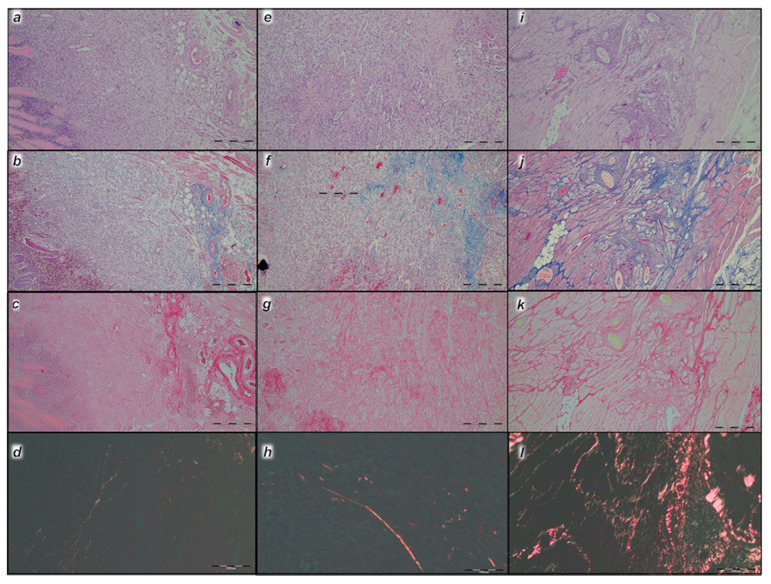
Illustrative presentation of the failed injury course in the control rats (***small italic bold letters***) on week 1 (***a***–***d***), week 2 (***e***–***h***), and week 3 (***i***–***l***) after surgical detachment of the quadriceps muscle from its attachments. Histochemical staining of bone–muscle lesion area after proximal rectus femoris muscle injury dissection. (HE staining (***a***,***e***,***i***), Masson trichrome staining (***b***,***f***,***j***), Sirius red staining (***c***,***g***,***k***), and Sirius red staining with polarized microscopy (***i***,***j***,***l***) (magnification 200×, scale bar 200 µm).

**Figure 20:**
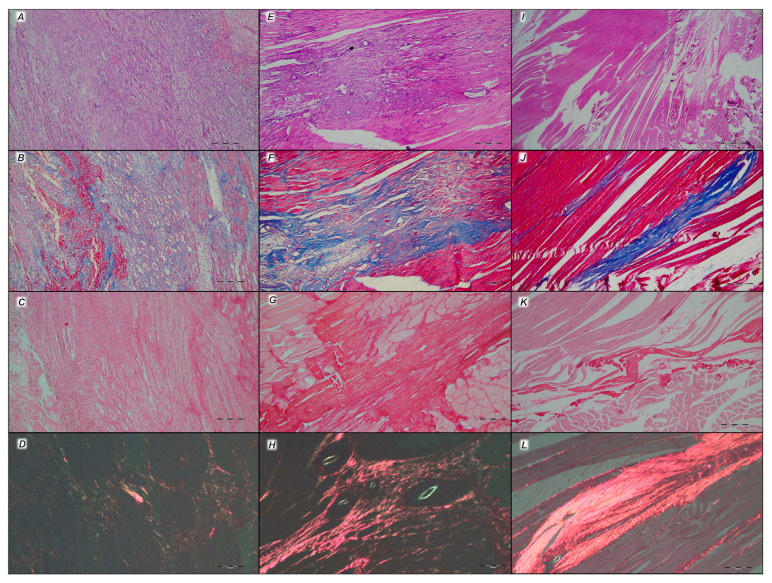
Illustrative presentation of the healing improvement in the injury course in the BPC 157-treated rats (***capital italic bold letters***) on week 1 (***A***–***D***), week 2 (***E***–***H***), and week 3 (***I***–***L***) after surgical detachment of the quadriceps muscle from its attachments. Histochemical staining of bone–muscle lesion area after proximal rectus femoris muscle injury dissection. (HE staining (***A***,***E***,***I***), Masson trichrome staining (***B*,*F*,*J***), Sirius red staining (***C*,*G*,*K***), and Sirius red staining with polarized microscopy (***I*,*J*,*L***) (magnification 200×, scale bar 200 µm).

Collagen proliferation on Sirius red staining was significantly increased in the treated animals compared to the controls at all three time intervals, 1, 2, and 3 weeks after injury (Figure 19 and Figure 20).

Polarized light microscopy imagery demonstrated the maturation of the newly formed collagen fibrils in the BPC 157-treated animals (Figure 20), which is in contrast with the control group, with minimal or no production of fibrils in the control group at the 3rd week (Figure 19). The results indicate the significantly increased production and appropriate orientation of collagen type 1 in the BPC 157 rats (Figure 19 and Figure 20).

Likewise, in accordance with functional and gross clinical assessments, the microscopic assessments demonstrated that BPC 157 therapy favors an increased rate of wound healing as well as reconstruction and orientation of collagen fibers (Figure 19 and Figure 20).

Specifically, in the 1st week, there was an infiltration of inflammatory cells, whereas the controls presented prominent edema, with no proliferation of collagen fibers (Figure 19). In the BPC 157 rats, there was an increased rate of proliferation of fibroblasts, with the synthesis of collagen fibers making loose connective tissue with mesh-like fibers and areolar tissue (Figure 20).

In the BPC 157 rats in the 2nd week, compared to the controls (Figure 19 and Figure 20), a moderate synthesis of collagen type 1 fibers was visualized using polarized microscopy Sirius red staining (Figure 20). In the controls, there were no significant changes compared to the 1st week; still, the infiltration of inflammatory cells and moderate edema were found (Figure 19), with a delay in fibroblast proliferation and the production of collagen fibers making loose connective tissue with mesh-like fibers and areolar tissue. The fibers were far enough apart, leaving ample open space for interstitial fluid in between. Although the fibers were strong enough to bind different tissue types together, they were still soft enough to provide flexibility and cushioning (Figure 19). Sparse and poorly-organized collagen type 1 fibers beginning in the 2nd week were observed in the controls (Figure 19). At the same time, edema almost completely vanished in the BPC 157 rats (Figure 20).

In the 3rd week, in the BPC 157-treated rats, prominent fibroblast proliferation with well-orientated collagen fibers synthesis was observed (Figure 20). There was a maturation of collagen fibers, as easily seen under Masson trichrome and Sirius red staining. Optimal orientation due to the long axis of the myofibers close to the bone and injury area with a lesser number of fibroblasts and a greater amount of collagen fibers was essential for the preservation of myofibrils, so no muscle fiber atrophy was noted. On the other hand, in the controls, revascularization vanished, and the proliferation of fibroblasts and fibers, as well as fiber maturation, was present but not to the extent seen in the BPC 157-treated rats. Fiber orientation was not optimal, due to the long axes of the myofibers close to the bone and injury area (Figure 19). Additionally, due to clinical contraction and spared use of the injured leg, muscle fiber atrophy occurred (Figure 19).

#### 3.6.3. Quadriceps Tendon

After surgical detachment of the quadriceps muscle from its bone attachments, quadriceps tendon disability presentation revealed the failed tendon injury course in the control rats, showing tendons with poor healing, along with the spontaneous inability to recover the muscle to the bone attachment to any extent. This failed tendon point was fully reversed in all the BPC 157-treated rats, along with recovering muscle-to-bone attachment (Table 1).

In summary, this study challenged the injured detached quadriceps muscle and muscle-to-bone reattachment in rats. We evidenced the recovery effect of the stable gastric pentadecapeptide BPC 157 therapy given orally [11,12,13,14,24,25]. These findings consistently support a tailored surgery regimen that includes adequate points, such as the dissection of the quadriceps muscle from its attachments to the femur and iliac bones, the complete dissection of the rectus muscle, and the partial dissection of vastus muscles at their proximal attachments. Likewise, the therapy effects of both BPC 157 regimens (i.e., µg- and ng- given in drinking water) were consistent, as evidenced by the macro/microscopic, ultrasonic, magnetic resonance, biomechanical, and functional assessments, resulting in muscle–bone reattachment.

## 4. Discussion

As previously suggested [11,12,13,14,24,25], the novel findings of muscle-to-bone reattachment after quadriceps muscle detachment (using macro/microscopic, ultrasonic, magnetic resonance, biomechanical, and functional assessments) using BPC 157 application theoretically and practically revealed that native peptide per-oral pharmacotherapy resolved muscle-to-bone reattachment. The compelling impacts of this effect were the quadriceps muscles detachment’s complex targets being resolved and the widely used regimen’s wide range of applied doses (µg-ng) supporting each other’s effects. The new therapy resolves the point of complete surgical detachment (rectus muscle) and partial surgical detachment (vastus muscles). Likely, such a consistent effect proves the concept by itself. Consistently, used per-orally, BPC 157 therapy overwhelms even the most threatful and complex conditions in rats after surgical detachment of the quadriceps muscles from their attachments, unlike permanent complete inability and no spontaneous healing after quadriceps muscle-to-bone detachment. As recently reviewed [11,12,13,14,24,25], the revealed muscle-to-bone reattachment likely interconnected particular wound-healing potential [14,25], the simultaneous healing of different tissues [11,12,13,14,24,25], recovery of junction formation [13,26,27,28,29] (i.e., recovery of the severed myotendinous junction [13] in particular), detached and/or transected muscle [30,31,32,33,34], tendon [27,28,29,34,35,36,37], ligament [38], and bone [39,40,41] injury healing.

We note that the BPC 157 therapy, with the specific effect of reversing course, overwhelms the combined severity of temporal muscle detachment [18] and quadriceps muscle detachment. Together, spontaneous healing after 8 weeks means that temporal muscle detachment in monkeys represents only a mild course [18]. This course was overwhelmed by the quadriceps muscle detachment’s much more severe difficulty and markedly increased lesion severity (being unable to heal after 3 months). From the beginning, permanent spontaneous healing with complete inability (knee flexed, hip flexed, circumduction motion of the leg with pelvic tilting, permanent leg contracture, and failed walking) hindered healing. Soon, upon detachment, there was a huge gap between the muscle and bone (the ultrasonic assessment showed that the muscle could not approach the bone; microscopy showed significant separation of muscle from bone). There was a minimal periosteum reaction and few mesenchymal cells only after the end of the first week. Consistently, 90 days after detachment, there was loose connective tissue, and the muscle (i.e., many non-matured and only a few red fibers) was separated from the bone. Finally, having undergone major (regularly insurmountable) harm, the muscle was without close contact with the bone, and bone islets appeared within the connective tissue. Thus, these disturbances were consistent with and more severe than those previously reported [18]; the periosteum, vascular supply between the muscle and periosteum, and the interconnections between muscle and bone were damaged and disrupted; the direct effects of muscle tension and viscoelasticity were eliminated; new bone formation was eliminated; and an intervening connective tissue layer separated the muscle from the bone. In summary, these findings are consistent with regularly irreparable healing failure in quadriceps muscle-to-bone detachment.

However, the BPC 157 application fully reversed this detachment healing failure, acting as a rapid therapeutic effect toward muscle-to-bone reattachment. The BPC 157 therapy-reversing course acts from the beginning, reversing an otherwise insurmountable deleterious course, and all parameters of the walking pattern were fully improved. Soon after detachment and therapy application, muscle approached the bone, leaving a minimal gap (as determined by ultrasonic assessment); microscopy showed minimal muscle separation from bone, and leg contracture was annihilated. The healing process occurred immediately after detachment from both sides: the muscle and the bone. The reattachment fibers from the ends of the muscle could be traced into the new bone formed at the surface (note, on day 3 post-detachment, there was an increase in mesenchymal cells with periosteum reactivation). Consequently, 3 months following detachment, there was well-organized bone, which was newly formed as more cortical bone due to the narrower bone marrow space; the muscle and mature fibers were oriented parallel with the bone axis and in close contact with the bone. The reversal of the injurious course using therapy fully organized muscle-to-bone reattachment, providing stability of form, and the balance between the muscle and bone was re-established.

The BPC 157 therapy-reversing course is an interconnected mode of healing, sharing commonalities that mutually support each other’s effect [11,12,13,14,24,25]. Presenting as an adequate compensatory capacity, the preserved quadriceps tendon myotendinous junction [13] and preserved part of the muscle (i.e., vastus muscles) take the function of the whole muscle (i.e., injured hind limb contracture disappeared, and the dissected quadriceps muscle instantly approached its bone attachments). Vice versa, this ascertained rapid function recovery of the detached muscle (i.e., completely detached rectus muscle and partly detached vastus muscles). Accordingly, the commonly observed disappearance of the contracture of the injured leg after BPC 157 therapy (recovered in muscle [30,31,32,33,34], tendon [27,28,29,34,35,36,37], and ligament [38] injury) illustrates recovery after quadriceps muscle detachment (Figure 5), especially detailed (as before) in the severed myotendinous junction and recovery [13]. Without therapy, the injured rats maintained knee flexure, with no knee extension even upon forced extension, awake or anesthetized, or immediately after their death [13]. Furthermore, as a common point, rats with severed myotendinous junctions [13] and rats with muscle-to-bone detachment shared in their recovery the same longitudinal collagen fibers’ orientation (note that the maturation of collagen fibers was seen under Masson trichrome and Sirius red staining). This should be able to recover muscle and tendons within the same time period [13], as well as muscle and bone in the muscle-to-bone reattachment. With BPC 157 therapy, the careful dissecting of the quadriceps tendon from the quadriceps muscle [13] and quadriceps muscle detachment from its attachments both resulted in muscle junction recovery (i.e., myotendinous junction as muscle-to-bone junction). Likewise, as a common point, there was a shared annihilation of the intervening connective tissue layer that separates the muscle from the bone (muscle-to-bone detachment) or muscle from the gap area (transected muscle) [30] and shared purposeful muscle cell proliferation. Day 4 following quadriceps muscle transection showed the muscle cells proliferating from both the proximal and distal sides in the lateral borders of the gap to the defect center [30]. Accordingly, in muscle-to-bone reattachment, there were mature fibers, oriented parallel with the bone axis and in close contact with bone. Thus, in the BPC 157 rats during post-transection healing [30] and post-muscle-to-bone detachment healing, full function restoration meant that the regenerating myofibers were not impeded by connective tissue. Finally, there were no bone islets within the connective tissues in the BPC 157 rats following muscle-to-bone detachment, muscle transection, tendon transection, and ligament transection [27,28,29,30,31,32,33,34,35,36,37,38]. Bone islets within connective tissues following muscle-to-bone detachment, muscle transection, tendon transection, and ligament transection [27,28,29,30,31,32,33,34,35,36,37,38] could be found, along with bone islets as a common finding [68]. Thus, it is likely that with BPC 157 therapy, no extraosseous bone islet means the counteraction of the endochondral bone formation in extraskeletal tissues, the counteraction of vascular endothelial cells differentiation into skeletal cells, local inflammatory signals, and the release of cytokines (i.e., bone morphogenetic protein-2 and bone morphogenetic protein-4 and transforming growth factor-beta) [68]. Likely, BPC 157 therapy is an orchestrated process that generally avoids or resolves impeding dysregulation processes.

Consequently, this suggests that all of the emphasized failure points (i.e., failed blood flow, osteoblast proliferation, and muscle tension) should be resolved, so that muscle-to-bone healing can be ongoing [18]. These were accordingly resolved throughout the given BPC 157 therapy. As pointed out [18], blood flow recovery was adequate, given osteoblast proliferation and new bone formation in the BPC 157 rats. Providing that muscle reattachment was realized accordingly with new bone initiation (i.e., post-detachment day 3), the direct effect of muscle tension was, at least partly, re-established [18]. This may be a beneficial effect (“bypassing key”), resolving a wide range of occlusion/occlusion-like syndromes vessels and multiple organ failures (for review, see [24]) by re-establishing blood flow through the activation of the collateral additional vascular pathways. This occurred immediately upon the application of BPC 157 therapy in rats with occlusion/occlusion-like syndrome with permanent major vessel occlusion and similar noxious procedures that all severely affect endothelium function [24]. Therefore, it acts from the beginning and reverses otherwise insurmountable deleterious courses in rats with detached quadriceps muscle from their attachments at the ilium and femur. Finally, BPC 157 therapy (according to Fourier transform infrared spectroscopy evidence) is tightly interconnected with the increased capability of a vessel to function, even in the worst circumstances [69]. These are the rapid changes in the lipid contents and protein secondary structure conformation in the vessel wall, which appeared very rapidly and were produced instantly by BPC 157 application [69]. Note, as recently reviewed [24], that stable gastric pentadecapeptide BPC 157 therapy might rapidly functionally enable minor vessels to take over the function of disabled major vessels, reorganize blood flow, and compensate for failed vessel function.

Moreover, for muscle-to-bone reattachment, BPC 157 therapy has had its special muscle healing and function recovery reviewed in detail (“muscle–brain axis”), specifically recovering and maintaining the function and integrity of the striated, heart, and smooth muscles, along with the recovery of the prime injury and muscle weakness as part of the peripheral and central effect (for review see [11,12]). As emphasized [11,12], counteraction occurred as a general shared effect, given the counteraction of the muscle weakness with abdominal aorta anastomosis, stroke, traumatic brain injury, spinal cord compression, neuroleptic catalepsy, amphetamine hypermobility, hyperkalemia, hypokalemia, hypermagnesemia, hyperlithemia, insulin hypoglycemia, local anesthetics, neurotoxin (parkinsogenic 1-methyl-4-phenyl-1,2,3,6-tetrahydrophyridine (MPTP), multiple sclerosis-like cuprizone), and tumor-induced muscle cachexia [11,12]. Likewise, BPC 157 interacts with the systems implicated in muscle functioning, dopamine, serotonin, acetylcholine, GABA, and glutamate [70,71]. Accordingly, the regained muscle-to-bone reattachment can be linked to a particular muscle–brain circuit to accommodate coordinated action on the quadriceps muscles [11,12]. In this, for the organization of the brain–gut axis, the specific neurotransmitter or neurotransmitter-like role of BPC 157 was suggested [70,71].

Thus, such healing effects in practical realization (i.e., muscle-to-bone reattachment, macro/microscopic, ultrasonic, MRI, biomechanical, and functional, via per-oral way) mean more advanced strategies. These overwhelm standard growth factors, as all are rapidly destroyed in human gastric juice within 15 min [72], are unable to be applied alone, and require the addition of various carriers or biological scaffolds or various combinations (for review, see [14]). Consequently, these inabilities of growth factors are practical obstacles that could preclude suited determination of the underlying general mechanisms that could back the obtained findings (for review see [14]). Thus, with such general limitations [14], skipping its interaction with many molecular pathways [36,37,43,44,45,46,47,48,49,50,51] (in particular, improved anabolic and counteracted catabolic pathways implemented in the recovery of the tumor-induced muscle cachexia [51]), the sustained, safe, and reproducible delivery of the BPC 157 is effective outside general peptide terms. As pointed out before [71] based on the pharmakokinetics study in the rat after single oral administration carried out in Istituto di Ricerche Biomediche “A. Marxer”, a half-life of BPC 157 is 66 h in male rats and 69 h in female rats.

Consequently, in muscle-to-bone reattachment, it could be that BPC 157 is given intragastrically (initially), and then per-orally (in drinking water as stable in water for a long time); it is native and stable in human gastric juice and is, thereby, transported from the stomach, giving a hormone-like effect [24]. Likewise, due to the large presence in human fetuses and adult tissues (in situ hybridization and immunostaining) [14], the concept holds a regulatory physiologic role in bodily functions [14], also based on similar beneficial effects in other species (i.e., birds [73] and insects [74,75,76]). This would approach cytoprotective capabilities, where stomach epithelium/endothelium protection might be easily achieved and further extended to the general level (protection of other organs) (cytoprotection to organoprotection) [24]. For muscle recovery in particular, these might be specific effects in vascular studies (for review, see [24]), and the effect would ascertain blood flow recovery via collateral pathways activation along with the mentioned muscle function recovery. There is real proof of activity but only vague estimations of the unresolved underlying mechanisms; thereby, limitations of this only being a descriptive study should be further addressed in future research. This could possibly be carried out given its special interaction and modulatory effects with the nitric oxide (NO) system [43,44,45,77,78,79,80,81], thrombocytes [81,82,83], and many molecular pathways [36,37,43,44,45,46,47,48,49,50,51], particularly NO molecular pathways [43,44,45], which is a point also noticed in myotendinous junction recovery [13].

In conclusion, the practical relevance of quadriceps muscle-to-bone reattachment as a new therapy point is further supported by a very safe BPC 157 profile [11,12,13,14,24,25]. No adverse effects were noted in clinical trials (ulcerative colitis, phase II) [84,85], and in toxicological studies, a lethal dose (LD1) was not achieved (for review, see [11,12,13,14,24,25]). Other clinical findings are supportive [86,87,88], particularly considerable long-lasting knee pain relief, suggesting the potential to repair tears, build cartilage, and reduce the number of knee surgeries [88]. Recently, a large study by Xu and collaborators [89] confirmed this favorable point. Therefore, the native peptide therapy, with per-oral cytoprotective pentadecapeptide BPC 157, which is known to be stable in human gastric juice, could provide a beneficial effect in muscle and tendon and bone healing [11,14,24,25]; this can be effectively combined to re-establish muscle–bone reattachment. In this way, its effects, i.e., functional, ultrasonic, magnetic resonance, biomechanical, macroscopic, and microscopic effects, were unmistakably attributed, which we clearly demonstrated would also represent defined muscle–bone reattachment healing in further practice.

## Figures and Tables

**Table 1:** Healing improvement by BPC 157 therapy in tendon lesions assessment (score 0–9) Min/Med/Max, using collagen, vascularization, and cellularity assessment (score 0–3) in rats at 1 day, 2 days, 3, days, 5 days, 7 days, 14 days, 21 days, 28 days, 60 days, and 90 days following surgical detachment of the quadriceps muscle from its attachments.

The Injury Course After Surgical Detachment of the Quadriceps Muscle from Its Attachments	Medication	Tendon Lesions Assessment Min/Med/Max			
		Collagen Assessment (Score 0–3)	Vascularization Assessment (Score 0–3)	Cellularity Assessment (Score 0–3)	Total Score Assessment (Score 0–9)
1 day	Control	3/3/3	3/3/3	3/3/3	9/9/9
	BPC 157 10 µg	3/3/3	3/3/3	3/3/3	9/9/9
	BPC 157 10 ng	3/3/3	3/3/3	3/3/3	9/9/9
2 days	Control	3/3/3	3/3/3	3/3/3	9/9/9
	BPC 157 10 µg	3/3/3	3/3/3	3/3/3	9/9/9
	BPC 157 10 ng	3/3/3	3/3/3	3/3/3	9/9/9
3 days	Control	3/3/3	3/3/3	3/3/3	9/9/9
	BPC 157 10 µg	3/3/3	3/3/3	3/3/3	9/9/9
	BPC 157 10 ng	3/3/3	3/3/3	3/3/3	9/9/9
5 days	Control	3/3/3	3/3/3	3/3/3	9/9/9
	BPC 157 10 µg	3/3/3	*2/2/2* *	*2/2/2* *	*7/7/7* *
	BPC 157 10 ng	3/3/3	*2/2/2* *	*2/2/2* *	*7/7/7* *
7 days	Control	3/3/3	3/3/3	3/3/3	9/9/9
	BPC 157 10 µg	3/3/3	*2/2/2* *	*2/2/2* *	*7/7/7* *
	BPC 157 10 ng	3/3/3	*2/2/2* *	*2/2/2* *	*7/7/7* *
14 days	Control	3/3/3	3/3/3	3/3/3	9/9/9
	BPC 157 10 µg	*2/2/2* *	*2/2/2* *	*2/2/2* *	*6/6/6* *
	BPC 157 10 ng	*2/2/2* *	*2/2/2* *	*2/2/2* *	*6/6/6* *
21 days	Control	3/3/3	3/3/3	3/3/3	9/9/9
	BPC 157 10 µg	*1/1/1* *	*2/2/2* *	*2/2/2* *	*5/5/5* *
	BPC 157 10 ng	*1/1/1* *	*2/2/2* *	*2/2/2* *	*5/5/5* *
28 days	Control	2/2/2	3/3/3	3/3/3	8/8/8
	BPC 157 10 µg	*0/0/0* *	*1/1/1* *	*0/0/0* *	*1/1/1* *
	BPC 157 10 ng	*0/0/0* *	*1/1/1* *	*0/0/0* *	*1/1/1* *
60 days	Control	2/2/2	2/2/2	2/2/2	6/6/6
	BPC 157 10 µg	*0/0/0* *	*1/1/1* *	*0/0/0* *	*1/1/1* *
	BPC 157 10 ng	*0/0/0* *	*1/1/1* *	*0/0/0* *	*1/1/1* *
90 days	Control	2/2/2	1/1/1	1/1/1	4/4/4
	BPC 157 10 µg	*0/0/0* *	*1/1/1* *	*0/0/0* *	*1/1/1* *
	BPC 157 10 ng	*0/0/0* *	*1/1/1* *	*0/0/0* *	*1/1/1* *

Initial application of the regimen that was used in drinking water; BPC 157 10 µg/kg or 10 ng/kg (or water (1 mL/rat) (controls)) was given intragastrically at 5 min postoperative time. *—p ˂ 0.05, *at least* vs. *control*.

## Data Availability

The original contributions presented in the study are included in the article, further inquiries can be directed to the corresponding authors.
